# Nesting in anticipation: Spatial ecology of giant honey bees (*Apis dorsata*) in relation to crop succession mapped by remote sensing

**DOI:** 10.1371/journal.pone.0347045

**Published:** 2026-06-24

**Authors:** Gerald Kastberger, Wolfgang Sulzer

**Affiliations:** 1 Department of Biology, University Graz, Graz, Austria; 2 Department of Geography and Regional Science, University Graz, Graz, Austria; University of Alberta, CANADA

## Abstract

Seasonal migration and nesting of the giant honeybee *Apis dorsata* (Fabricius, 1793) are widely associated with mass-flowering crops, yet how colony settlement relates to surrounding landscape structure remains poorly understood. We analysed land-use patterns within the effective foraging range of *A. dorsata* colonies in the central Chitwan plain, Nepal, using satellite-derived land-use maps from three seasonal snapshots spanning one agricultural cycle: December 1999, February 2000, and April 2000. For each season, land-use composition was quantified radially around nesting sites and compared with spatially constrained random reference landscapes, allowing background seasonal turnover to be separated from nest-specific spatial contrasts. To evaluate both the magnitude and spatial consistency of these contrasts across colonies, we developed a signal–precision framework that integrates contrast strength with repeatability across 55 nesting sites, comprising approximately 300 nests, and 55 matched random locations. Across seasons, the strongest nest-associated signals corresponded to two nectar- and pollen-relevant crops: mustard in December 1999 and buckwheat in February 2000. These signals peaked within 1–3 km of nests, consistent with the effective foraging range of *A. dorsata*, whereas the distinction between nest-centred and random landscapes became less pronounced at larger distances. Although mustard and buckwheat covered smaller proportions of the landscape than dominant cereals, such as wheat and rice, or other land-use classes, including grassland and fallow land, they consistently emerged as the most spatially precise nest-associated components, highlighting their diagnostic relevance for nesting environments. By separating nest-specific signals from seasonal land-use change and quantifying both their strength and spatial consistency, our colony-centred, null-model-based framework reveals how large-scale agricultural structure becomes reflected in colony settlement patterns. Through interannual return to traditional nesting sites, colony fidelity interacting with structured agricultural dynamics generates distinct nest-centred signals for forage-relevant crops, producing a pattern consistent with apparent anticipation of future resource availability. Although based on data from 1999–2000, this study provides a historical baseline of colony–landscape relationships, with ongoing work aimed at evaluating their persistence under current conditions.

## Introduction

Giant honey bees of the subgenus *Megapis* are widespread across South and Southeast Asia and include two species: the mainland giant honey bee *Apis dorsata* [1–4] and the Himalayan cliff bee *A. laboriosa* [1,5,6]. These hornet-sized bees construct large, single-comb nests in the open, attached to tree branches, cliffs, buildings, or other exposed structures. Colonies may occur singly, be widely dispersed across landscapes, or form massive aggregations of up to several hundred nests at a single site. A defining feature of both species is seasonal migration, typically occurring twice per year [7–9]. Colony-level site fidelity in *Apis dorsata* appears to depend on the queen’s comparatively long lifespan, allowing colonies to return to traditional nesting sites over multiple years [7,10].

Despite these observations [4,11–15], the ecological drivers and spatial organisation of migration in giant honey bees remain incompletely understood. Although migration in *Megapis* has been widely attributed to seasonal variation in floral resource availability [4,14,16–19], direct landscape-scale evidence linking migration timing, nesting-site distribution, and spatio-temporal resource dynamics remains scarce. Occasional but unconfirmed reports from parts of Lower Assam [20] suggest that *A. dorsata* nests may be present at the same sites over much of the year. This should not be taken to imply permanent residency of the same colony, but rather repeated nest occurrence at traditional sites under mild climatic conditions and prolonged floral resource availability. These observations raise the question of whether migration is driven primarily by specific mass-flowering resources, by landscape structure and heterogeneity, or by their combined influence.

**Fig 1:**
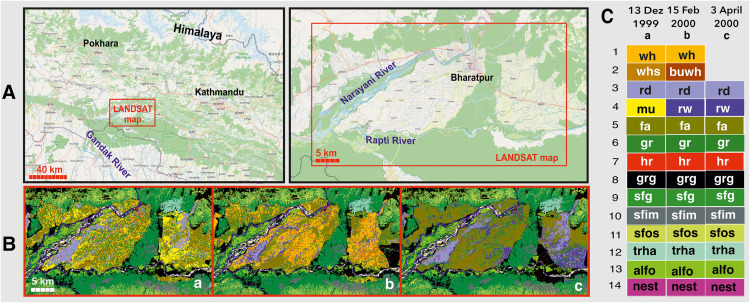
Land-use survey across three stages of the main colony spread of Apis dorsata in the Chitwan plains. A, Regional overview showing the Himalayan foothills and the southern Himalayan valleys from Pokhara to Kathmandu, extending from the Himalayan ridge to the Gangetic plains. Red rectangles indicate the positions of the three LANDSAT scenes used for the subsequent land-use classifications. B, Land-use classification based on LANDSAT imagery at three stages of the main colony spread in the Chitwan plains: (a) 13 December 1999, approximately one month after colony re-establishment and coinciding with the peak mustard cultivation period; (b) 15 February 2000, corresponding to the period when colonies reached their maximum size and reproductive activity, coinciding with the peak of buckwheat cultivation; (c) 3 April 2000, after mustard and buckwheat had disappeared from the crop rotation and following the main departure of colonies from the plains. C, Colour scales indicating the land-use categories used in the classification, based on a pixel resolution of 15 × 15 m, shown separately for each of the three survey stages **(a–c)**. Panel A is derived from OpenStreetMap data, and panel B from LANDSAT imagery (USGS/NASA, public domain); both were processed by the authors.

Here, we integrate field-based colony mapping with spatially explicit remote-sensing analyses [8,21,22] to investigate how land-use dynamics shape the nesting environments of *A. dorsata*. We focus on the Chitwan district of southern Nepal [8,15], a subtropical lowland region characterised by high colony densities and pronounced seasonal crop rotations. In this region (Fig 1), colonies migrate from surrounding forests into the central basin in late autumn, establish reproductive nests during winter, and depart again prior to the onset of the monsoon season. We analyse a uniquely detailed dataset from 1999–2000 that combines field-based nest censuses with satellite-derived land-use data. Although historical, this dataset captures colony–landscape relationships at a spatial resolution rarely available for large-scale pollinators and provides a baseline against which contemporary changes can be evaluated.

Rather than assessing land use at regional or administrative scales, we adopt a nestsite-centred, distance-resolved perspective, quantifying land-use composition as experienced by colonies within their biologically relevant foraging range. This approach explicitly links spatial scale, landscape heterogeneity, and colony distribution—three elements rarely integrated in studies of giant honey bee migration and nesting ecology [7,11,14,23,24].

We mapped approximately 300 *Apis dorsata* colonies at 55 nesting sites across an area of 1,380 km² in the central Chitwan plain (Fig 1), bounded by the Narayani River at Meghauli in the west (84° 7’3.70"E), the Rapti River in the south (27°28’26.64"N), the Narayani River near Bharatpur in the north (27°44’22.50"N), and east of the Tikauli Forest (84°38’41.84"E). Land use was classified from satellite imagery at three key stages of a single seasonal cycle: December 1999, approximately one month after widespread colony arrival and nest establishment; February 2000, coinciding with peak colony-level reproductive activity; and April 2000, following the main departure of colonies toward pre-monsoon roosting sites [15]. This design captures colony arrival, peak activity, and departure within one continuous migratory episode.

By integrating these temporally resolved LANDSAT scenes [8,21,22] with colony-centred spatial analyses, we examined how nesting-site distribution relates to the spatio-temporal dynamics of cultivated crops and associated land-use elements. Rather than inferring habitat preference or behavioural choice, our objective is to diagnose landscape components that consistently co-occur with nest locations under specific seasonal conditions. To this end, we introduce a signal–precision framework that jointly evaluates the magnitude of nest–random land-use contrasts and their spatial consistency across nesting sites.

By explicitly separating seasonal land-use dynamics from nest–random spatial structure, this approach provides a quantitative basis for characterising landscape features associated with nesting of *Apis dorsata* across different phases of the migratory cycle. Using Chitwan as a model system, our temporally resolved analysis shows how seasonal crop succession, landscape structure, and spatial scale jointly shape migration timing and nesting-site distribution. More broadly, the study introduces a null-model–based framework for diagnosing nest-associated landscape structure in seasonally dynamic agricultural systems, with potential relevance for landscape ecology and pollinator research.

## Materials and methods

### Study area

Fieldwork was conducted from February 17 to March 16, 2000 in Chitwan District, south-central Nepal, within the Inner Terai valley. This west–east oriented tectonic depression is bounded by the forested Churia foothills to the south and the Mahābhārat Range of the Siwalik Mountains to the north. The study was based on non-invasive field observations and spatial analyses. Access to the relevant field sites was obtained with the agreement of landowners / local authorities / local communities / site managers. No specific formal permit was required for this work under the regulations applicable at the time of the study.

LANDSAT-based analyses were restricted to the central plains of Chitwan, covering approximately 1,380 km² (Fig 1A). Although the broader district spans elevations of 130–1,350 m above sea level, the present analysis focuses exclusively on the lowland Inner Terai plain (mean elevation ~280 m); upland areas were excluded.

The area is structured by two major river systems: the Narayani River, flowing from northeast to southwest and forming the western and northern boundaries, and the Rapti River, flowing east–west along the southern margin.

Land use in the Chitwan plains is strongly shaped by human activity. Former wetlands have largely been converted into farmland, with rice, maize, mustard, wheat, and legumes cultivated in both simultaneous and rotational schemes. Pastures and grazed forest patches persist locally, but forest cover within the central agricultural plains is highly fragmented. In contrast, extensive forest areas are preserved south of the Rapti River within Chitwan National Park, a major protected area supporting high biodiversity.

### Remote sensing and land-use classification

**Fig 2:**
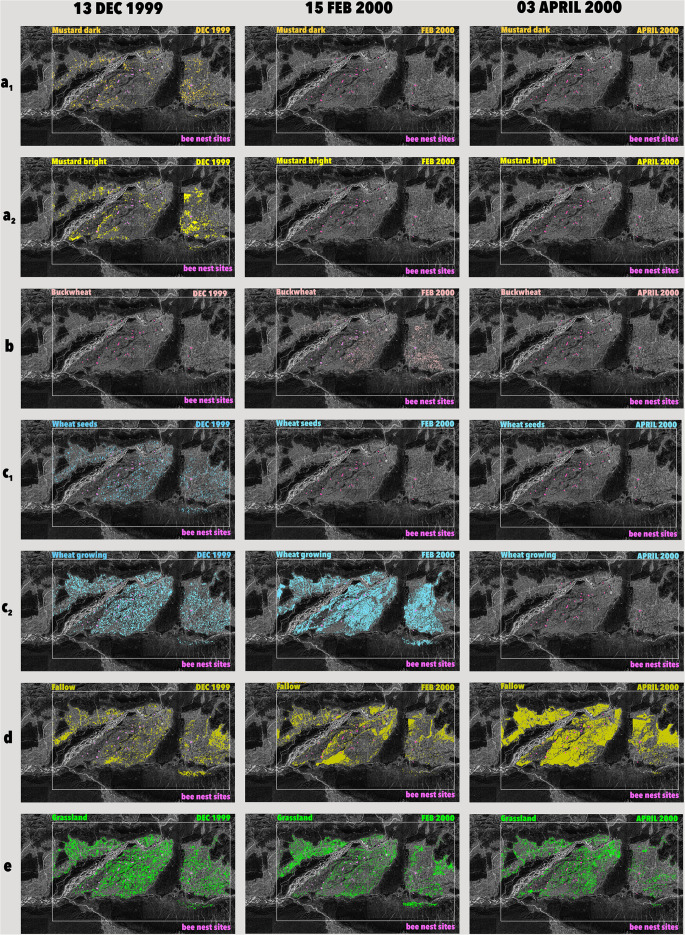
Spatial distribution of land-use components around nest sites across seasons. Nest-centered land-use overlays for the central Chitwan plain shown for three survey dates (13 December 1999, 15 February 2000, and 3 April 2000). In the figure layout, columns represent survey dates, and rows represent individual land-use components, displayed as color-coded overlays on a common grayscale base image. White rectangles indicate the analyzed landscape window surrounding nest sites. The depicted land-use categories are as follows (see color coding within panels): a₁–a₂, mustard (December-dominant mass-flowering crop of nutritional relevance to *Apis dorsata* colonies); b₁, buckwheat (February-dominant mass-flowering crop); c₁–c₂, wheat; d₁–d₂, rice; e, grassland (the remaining land-use elements are shown in S5f–S5k Figs). Wheat cultivation (panels c) extended across most of the plains during winter but was completely harvested by early spring, resulting in a marked increase in fallow land thereafter (S5f Fig). Rice fields (panels d_1,2_) were spatially restricted, occurring primarily along the Narayani River and east of the Tikauli forest. Mustard is confined to the December scene (panels a_1,2_), whereas buckwheat appears only in February (panel **b)**. Derived from LANDSAT imagery (USGS/NASA; public domain), processed by the authors.

Land-use dynamics in the central basin of the Chitwan plains were analysed using LANDSAT ETM+ satellite imagery [8,21,22] acquired at three key stages of the annual migration cycle of *Apis dorsata*: 13 December 1999 (post-arrival), 15 February 2000 (peak colony distribution), and 3 April 2000 (post-departure) (Figs 1,2).

All figures derived from these satellite data are analytical products based on LANDSAT imagery (USGS/NASA; public domain), generated by pixel-wise classification and re-analysis.

The three scenes represent key phases of the seasonal stay of *Apis dorsata* colonies. The December scene captures mustard flowering shortly after colony arrival, the February scene corresponds to peak reproductive activity associated with buckwheat flowering, and the April scene reflects post-harvest conditions when colonies had begun to leave the plains or redistribute into surrounding forest areas. While crop timing may vary interannually, these scenes represent the dominant agricultural sequence during the study period and are therefore treated as representative temporal snapshots.

The 1999–2000 dataset is used as a historical baseline rather than a proxy for present-day conditions. Its relevance lies in the integration of field-based nest census data with seasonally matched satellite imagery. The nest-centred versus random comparison was designed to identify spatial associations between colony placement and the seasonal land-use mosaic. We therefore interpret the results as evidence for colony–landscape coupling during the study period, while recognising that contemporary data are required to assess persistence. Current validation is underway using renewed field surveys and higher-resolution satellite imagery.

This remote-sensing approach enabled landscape-wide mapping across areas that were seasonally inaccessible or difficult to survey using field methods. Satellite analyses were complemented by targeted field observations for interpretation and validation.

### Image preprocessing and spatial integration

Geometric correction and terrain preprocessing were based on 1:25,000 topographic maps, from which a Digital Terrain Model (DTM) was generated. The DTM represents surface elevation independent of vegetation and built structures and was used to correct distortions caused by terrain relief and sensor geometry during orthorectification.

Historical land-use maps (1:50,000; Topographical Survey of Nepal, 1984) served as ancillary reference data. All spatial datasets—including GPS-based nesting-site coordinates and colony counts—were integrated into a Geographic Information System (GIS).

The December 1999 scene was geometrically corrected using 79 ground control points. The February and April scenes were co-registered to this reference via image-to-image rectification to ensure precise spatial alignment. Panchromatic and multispectral bands were combined by pan-sharpening, yielding a spatial resolution of 15 × 15 m.

### LULC classification approach

Land-use and land-cover (LULC) maps were generated using a hybrid classification strategy, as no single automated method reliably separated all ecologically relevant classes across seasons.

Rule-based classification was applied to spectrally distinct features (e.g., water bodies, river gravel, clouds), whereas supervised and unsupervised classification was used for heterogeneous land-use types. Manual digitisation was applied where automated separation was unreliable, particularly for cloud shadows.

Clouds were detected by thresholding, whereas their shadows required manual delineation to prevent confusion with swamps due to similarly low reflectance.

Forests were first delineated using a scene-independent mask and subsequently subdivided into major forest types using multispectral data and DTM-derived variables. This approach reduced confusion between forested and agricultural areas.

In the April 2000 scene, areas of reduced vegetation vitality within Sal forest were identified by lower values of the Normalized Difference Vegetation Index (NDVI).

Natural land-cover types such as gravel bars and grasslands were identified using rule-based classification combined with contextual refinement. Urban areas could not be reliably classified due to spectral mixing and limited spatial resolution; settlement and road features were therefore extracted from topographic maps and incorporated as vector layers.

All classified layers were integrated within the GIS to produce temporally consistent LULC maps forming the basis for quantitative nest-centred landscape analyses.

### Agricultural land classification

Vegetation phenology was characterised using NDVI and Tasseled Cap transformations, which summarise multispectral information into ecologically meaningful components related to vegetation greenness, surface brightness, and moisture.

Agricultural land was subdivided into phenologically distinct crop classes using a training-area-based maximum likelihood classifier, including mustard, buckwheat, wheat, rice, grassland, and fallow land. Training areas were derived from georeferenced field observations collected between 17 February and 16 March 2000, restricted to spatially homogeneous patches consistent with the LANDSAT resolution.

For the December and April scenes, where direct ground-truth data were not available, training areas were identified using a reproducible workflow integrating spectral similarity across dates, crop phenology, historical land-use maps, and high-resolution imagery.

To reduce pixel-level noise, pixel-based classification was complemented by object-based post-processing, in which spatially contiguous segments were assigned the dominant class. This yielded spatially coherent agricultural units suitable for landscape analysis.

### Colony-centred landscape analysis

Land-use composition around each nest was quantified using concentric rings centred on the nest at 15 m radial intervals, corresponding to the spatial resolution of the LULC maps. For each radius, pixels were classified by land-use category and aggregated across all 55 nests.

When pixel counts are summed across nests, totals exceed those of a single ring because counts are calculated separately for each nest and then aggregated. These totals follow the expected power-law scaling with minor systematic deviations arising from raster–geometry effects when circular buffers intersect square pixels. The high goodness of fit (R² > 0.996) confirms the robustness and reproducibility of the sampling procedure.

For each nest site, land-use proportions were calculated in concentric buffers extending up to 3000 m. Mean values were computed across rings for each land-use category, and grouped into frequency classes for visualisation as heat maps (Fig 4). Colour scales were defined separately for each category.

Circular sector plots (Fig 5) summarise land-use composition around individual nests, with angular width indicating proportional share and radial extent representing mean contribution on a logarithmic scale.

### Quantification of nest–random land-use contrasts

For each land-use category and radial distance, the difference in land-use representation between nest-centred landscapes and randomly placed reference landscapes was calculated as:


$$\begin{array}{c} {\Delta \text{LU}}_{\text{n}-\text{r}}=\;{\text{LU}}_{\text{n}}\;-{\text{LU}}_{\text{r}} \end{array}$$

where LUₙ and LUᵣ denote the proportional area of a given land-use category in nest-centred and random landscapes, respectively.

Random reference landscapes were generated within the study area under identical spatial constraints, forming an empirical null model within a Monte Carlo framework. This design ensures that comparisons are made within the same landscape context and avoids confounding by large-scale spatial gradients.

Spatial consistency of ΔLUₙ ₋ ᵣ across nesting sites was quantified as its standard deviation.

### Spatial coupling analysis

To assess relationships between land-use components, ΔLUₙ ₋ ᵣ values were calculated for concentric distance intervals up to 4.5 km. For each interval, values of individual land-use categories were plotted against those of mustard (December) and buckwheat (February), enabling identification of scale-dependent positive or negative associations.

### Crop rotation analysis

Crop-to-crop rotation was quantified by cross-tabulating land-use classes of identical 15 × 15 m pixels between December 1999 and February 2000. For each nest, pixels within distance bands (100–4500 m) were assigned transition categories. Transition counts were normalised to total pixel numbers and averaged across nests, yielding proportional rotation values (mean ± SE).

### Contrast strength and spatial precision

(a) Contrast **strength**

Contrast strength (M₍c₎) represents a normalised measure of nest–random contrast magnitude (Equation 1):


$$\begin{array}{c} M_{c}=\frac{\text{1}-{log}_{\text{1}\text{0}}\left(rel\;\left(\frac{\text{1}}{\left(\Delta \;{LU}_{n-r}\right)\;\text{2}}\right)\right)}{max\left\lfloor \text{1}-{log}_{\text{1}\text{0}}\left(rel\;\left(\frac{\text{1}}{\left(\Delta \;{LU}_{n-r}\right)\;\text{2}}\right)\right)\right\rfloor } \end{array}$$

Here, ΔLUₙ ₋ ᵣ denotes the difference in relative land-use proportion between nest-centred and random landscapes. The term *rel* denotes scaling to the maximum value within the dataset, yielding a dimensionless quantity prior to transformation.

High M_c_ values indicate strong deviation from random expectation, whereas low values indicate weak or no contrast.

(b) **Spatial precision**

Spatial precision (Pₛ) quantifies the consistency of ΔLUₙ ₋ ᵣ across nesting sites (Equation 2):


$$\begin{array}{c} \;P_{s}=\frac{\text{1}}{s_{x}\left(\Delta \;{LU}_{n-r}\right)\;\text{2}} \end{array}$$

where sₓ denotes the standard deviation across nests.

High Pₛ values indicate low variability and thus high spatial consistency.

### Interpretation of signal–precision space

Points located toward the upper right of the signal–precision plots represent land-use categories for which ΔLUₙ ₋ ᵣ is both large and consistent across colonies. These identify land-use components that repeatedly co-occur with nesting sites.

Together, contrast strength and spatial precision highlight land-use components characterised by strong and spatially coherent associations, while down-weighting weak or heterogeneous patterns. This framework enables direct comparison among land-use types and facilitates identification of diagnostic landscape signals.

## Results

### Seasonal land use turnover and colony-centered foraging landscapes

In a broader perspective, land-use patterns within the foraging range of *Apis dorsata* colonies in central Chitwan differed strongly between December 1999 and February 2000 (Fig 1Ba,1Bb), consistent with seasonal crop rotation driven by farmers’ management decisions and prevailing environmental conditions. By April 2000 (Fig 1Bc), the previously distinct seasonal crop patterns had largely disappeared, as mustard, buckwheat, and wheat were absent from the land-use imagery and only rice remained as the dominant crop. Field observations from the same period suggest that by early March 2000 many colonies had already left the region, while others showed signs consistent with imminent absconding [11,17,25–28].

**Fig 3:**
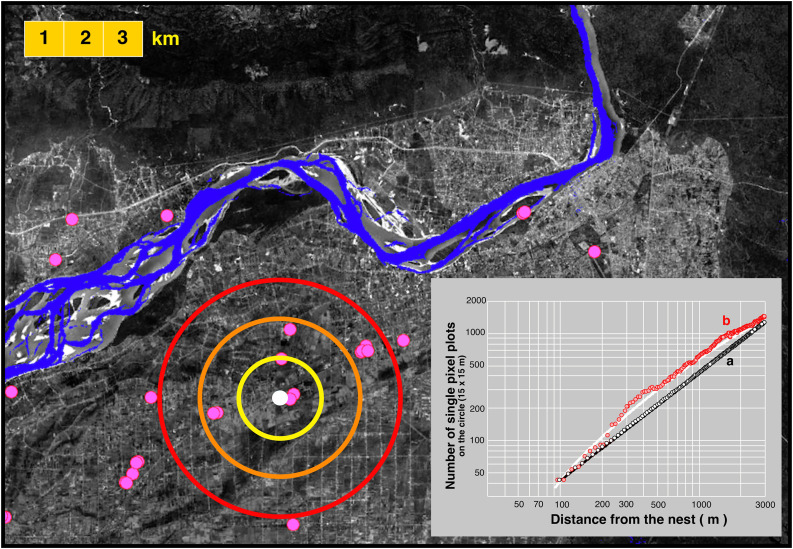
Definition of the spatial extent and nest-site–centered (“bee-eye”) perspective used for land-use analysis. The main panel shows a section of the classified land-use map centred on one of the nesting sites located on the Rampur campus (solid white circle). Purple spots indicate the positions of additional nesting sites identified in the surrounding area (cf. S2 Fig). Concentric circles (yellow, orange, red) indicate radii of 1, 2, and 3 km around the selected nesting site 38. The inset panel presents the corresponding quantitative analyzes, showing the number of LANDSAT pixels intersecting each individual concentric circle as a function of distance from the nest. Each LANDSAT pixel represents an area of 15 × 15 m, and circular assessments were performed at radial increments of 15 **m.** Curve (a) shows the theoretical number of pixels contained within each discrete circular assessment around the nest (i.e., per radius, not cumulative). Curve **(b)** (solid red circles) depicts the number of identified land-use elements for the selected nest site 38 in the December 1999 LANDSAT imagery within the same circular assessments, classified into 13 distinct land-use categories. The deviation from the theoretical distribution does not primarily reflect double counting, but rather the geometric mismatch between circular buffers and square raster pixels. Lines indicate polynomial regressions fitted to the data (a: R² = 1.000; b: R² = 0.996). Derived from LANDSAT imagery (USGS/NASA; public domain), processed by the authors.

**Fig 4:**
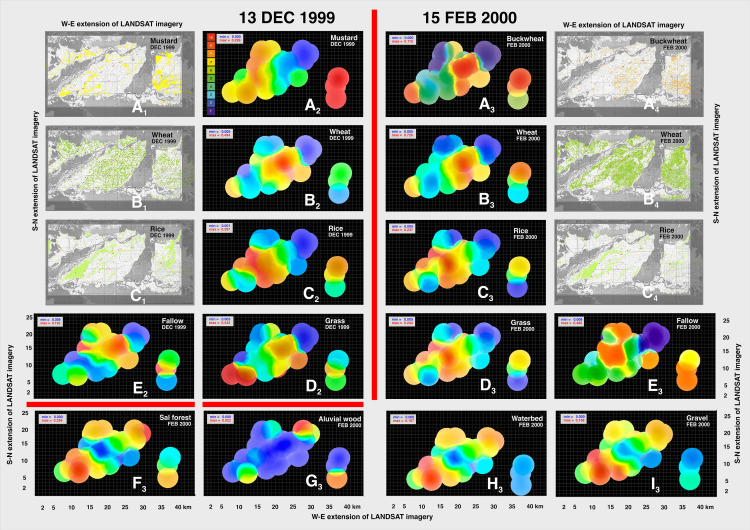
Nest-site specific land-use patterns in the central Chitwan plain in December 1999 and February 2000. Pixel-level land-use classification derived from satellite imagery is shown in panels 1 and 4, and corresponding colony-centered summaries (based on 55 nest sites) are shown in panels 2 and 3. For each nest site, land-use proportions within a 0–3,000 m radius (mean ± SE) were grouped into ten frequency classes and visualised as heat maps ranging from blue (low; land-use proportion = 0) to red (high; land-use proportion = 0.30). Standard errors were also calculated, but are not shown here; because the colour coding is based on relatively broad categories, the mean-based patterns remain clearly interpretable. The corresponding variability estimates are provided separately in the vector plots of Fig 5. Color scales span the observed crop-specific minimum–maximum frequencies. The background grids in panels 2 and 3 represent 1-km² cells covering 45 km **(W–E)** × 26 km **(S–N)**. Colony locations in panels 1 and 4 are marked in red (compare S2 Fig). In panels 2 and 3, circular color-coded sectors around each nest illustrate the surrounding land-use composition and the degree to which colonies are embedded in high-value crops (mustard, buckwheat) versus grassland, riparian, forested, or other semi-natural habitats. Land-use indices are scaled from 0 to 1; component-specific ranges for February 2000 are given in panels A_2_–I_2_. Panels 1–2 correspond to December 1999 (mustard, wheat, rice, grassland, fallow), and panels 3–4 to February 2000 (buckwheat, wheat, rice, grassland, fallow). The bottom row (Sal forest, alluvial forest, gravel, water) represents landscape-stable components that change little between seasons. Thick red bars distinguish December from February. Derived from LANDSAT imagery (USGS/NASA; public domain), processed by the authors.

To translate these seasonal land-use changes into a colony-relevant context, the landscape surrounding each nesting site was analyzed from a foraging-centered (“bee-eye”) perspective within a biologically realistic radius of up to 4.5 km. This approach complements the crop-specific, satellite-based landscape perspective (Fig 2; S2 Fig) by representing land-use patterns from a colony-centered foraging perspective. For each crop type, the frequency of occurrence around all 55 nesting sites was quantified as a function of distance from the nest center (Fig 3), from the immediate vicinity out to 4.5 km, and grouped into ten frequency classes (Fig 4).

### Spatial and seasonal contrasts in crop availability

The analysis displays primarily the western Chitwan plain, where most nesting sites are located. Three sites occur within agricultural zones east of the Tikauli forest corridor, and two lie north of the Narayani River, but all nesting sites included in this study are north of the Rapti River.

In the central Chitwan plain west of the Tikauli forest, mustard cultivation (Figs. 2a_1_, 2a_2_; Fig. 4A_2_) is concentrated mainly in the western sector, whereas east of this corridor mustard is largely absent and other land-use types prevail. In contrast, buckwheat in February 2000 (Fig 2b; Fig 4A_3_) is most abundant farther east in the central plain, occupying areas that are not dominated by mustard in December 1999. This east–west differentiation, evident even within the central plain, is consistently reflected in both the satellite-based classification (Fig 2; Figs 4A₁–4C₁; Figs 4A₄–4C₄) and the nesting-site-centered “bee-eye” perspective (Figs 4A2–4E2; Figs 4A3–4I3).

Only a single nesting site, located in the northeastern part of the study area near or within urban Bharatpur (see S2j Fig), is isolated from both mustard and buckwheat in both months. At this site, surrounding Sal and alluvial forests, together with nearby urban markets, are likely to provide alternative foraging resources.

Wheat is widespread across the landscape (Figs 2c_1_,2c_2_); however, from a nest-centered (“bee-eye”) perspective (Fig 4B), its spatial distribution is concentrated in the eastern plain in both December 1999 and February 2000, where it overlaps with buckwheat cultivation in February (Fig 2b; Fig 4A₃). Rice shows a contrasting pattern: from the colonies’ perspective (Figs 4C_2_,4C₃), it is most prominent in the central part of the plain, whereas the satellite perspective (Fig 2b; Figs 4C₁,4C₄) reveals a strong association with the Narayani River and its floodplain. Grassland (Fig 2e; Figs 4D₂,4D₃) and fallow land (S2f Fig; Figs 4E₂,4E₃) exhibit pronounced seasonal changes between December and April, consistent with crop cultivation and rotation involving wheat, rice, mustard, and buckwheat, as well as their subsequent harvest.

By contrast, Sal forest (S2g Fig; Fig 4F_3_), alluvial forest (S2h Fig; Fig 4G_3_), pine forest (S2i Fig), water bodies (S2k_1_ Fig; Fig 4H_3_), and gravel (S2k_2_ Fig; Fig 4I_3_) exhibit highly similar spatial distributions across seasons, reflecting their relative stability within the landscape. Although gravel occurs at low overall abundance, its spatial distribution closely parallels that of riverbeds (Fig 4H_3_) and Sal forest (Fig 4F_3_). Because these land-use components exhibit little seasonal variation, they are shown only for February in the nest-centered perspective (Figs 4A_3_-4I_3_).

### Nestsite-specific land-use composition

**Fig 5:**
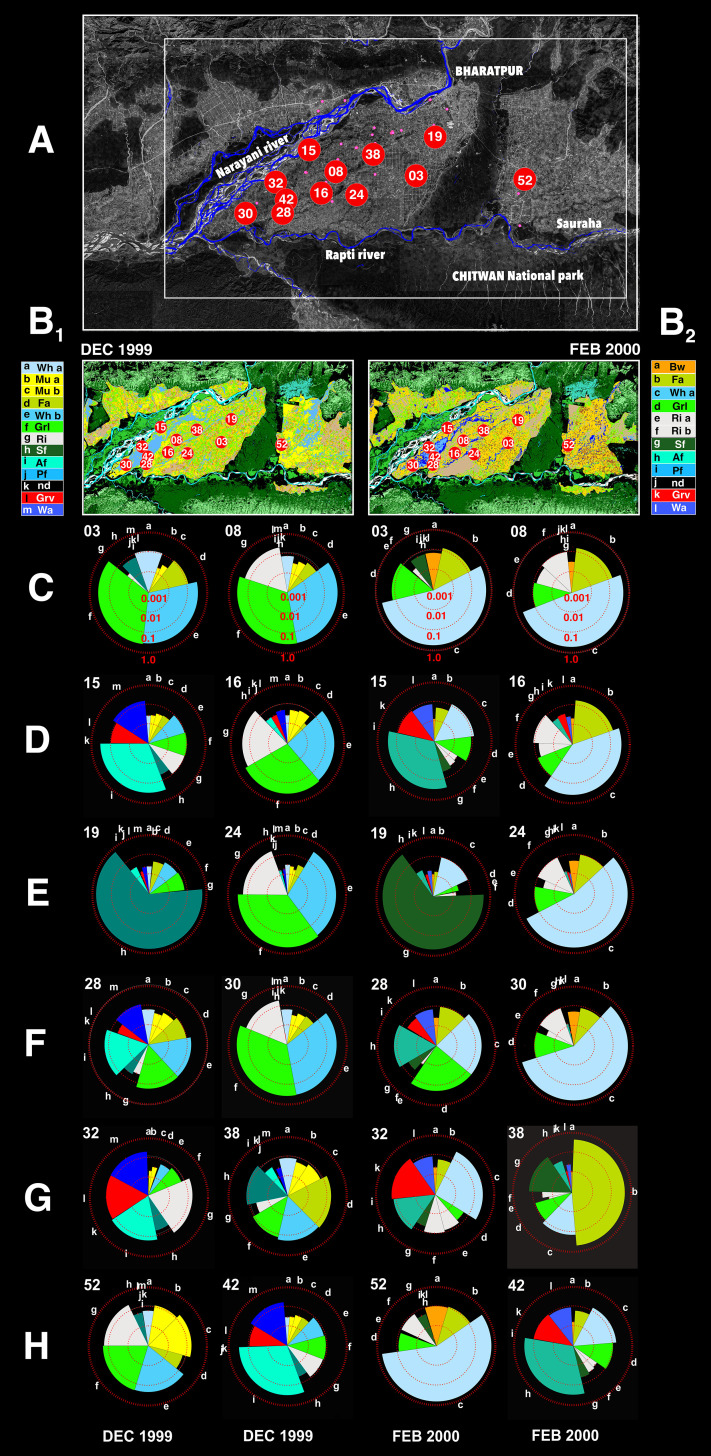
Seasonal comparison of nesting-site surroundings and directional land-use patterns at selected nest sites of Apis dorsata colonies in central Chitwan (December 1999 and February 2000). A, Overview of the study area in the Chitwan Valley, Nepal (red rectangle; ~ 48 km **(W–E)** × ~ 27 km **(S–N)**, showing the locations of 12 representative nesting sites (red circles). Numbers correspond to nesting-site identifiers. B, Land-use classification maps derived from satellite imagery for December 1999 (B₁) and February 2000 (B₂). Colors indicate the main land-use categories; class assignments differ slightly between months to reflect seasonal crop changes (mustard/buckwheat, wheat, rice, grassland, fallow, forests, gravel, water, and non-identifiable areas). **(C–H)** Twelve pairs of radial plots selected from the full set of 55 nesting sites, illustrating variability in the surrounding land-use landscape. For each nesting site, mean proportions of land-use classes are shown within a 4.5 km radius. For each nesting site, the surrounding landscape was sampled in azimuthal sectors of 22.5° and in concentric distance steps of 15 m from 100 to 4500 m radius. Within each sector and distance class, the prevailing land-use category was recorded. For each land-use element, mean proportions and associated standard errors were then calculated and are displayed here as radial vector plots. The mean proportion of each land-use class is shown here in a sector-shaped graphical element; its angular width and outer radial extent represent the mean value. Small red points at the outer boundary indicate the associated standard errors, calculated from the underlying azimuthal sampling sectors and 15 m concentric sampling rings. Each pair compares the same nesting site between December 1999 (left) and February 2000 (right). Concentric reference circles denote cumulative proportional coverage (0.001, 0.01, 0.1, 1.0). Derived from LANDSAT imagery (USGS/NASA; public domain), processed by the authors.

As a next analytical step, Fig 5 extends the crop-specific, colony-centered representation by integrating all mapped land-use components into circular diagrams for selected nesting sites. These diagrams summarize, for each nest, the composition of the surrounding foraging landscape and thereby provide a nest-site-specific landscape signature showing how different land-use components co-occur, irrespective of their direct relevance as forage. Each circle displays the relative contribution of all mapped land-use categories around a single nest site, grouped into ten frequency classes and arranged radially to allow direct comparison across sites and seasons. In contrast to the crop-specific heat-map perspective (Fig 4), this integrated representation highlights pronounced among-site heterogeneity in land-use composition, even within the same season.

While some nest sites are embedded in landscapes dominated by one or two major crop types, others occur in more heterogeneous mosaics in which several land-use categories contribute at intermediate frequencies. Such differences are not apparent when crops are considered separately but emerge clearly when all components are integrated at the nest site level. Notably, among the land-use components shown, mustard and buckwheat represent the primary forage-relevant crops for the colonies. However, both occur at relatively low frequencies and were not consistently present across sites or seasons in the Chitwan landscape (Figs 2a,2b).

### Distance-dependent nest–random contrasts in seasonally shifting landscapes

Seasonal comparison reveals pronounced shifts in nest-specific landscape signatures between December 1999 and April 2000 (Figs 1a–1c), as changes in mass-flowering crop (mustard, buckwheat) dominance are offset by a combination of actively managed crops (wheat, rice) and passively emerging land-use states following harvest (grassland and fallow). Consequently, colony-level landscape composition (Figs 4, 5) reflects the integrated contribution of multiple land-use types rather than dependence on a single crop, consistent with seasonal agricultural management and crop rotation during winter 1999/2000.

**Fig 6:**
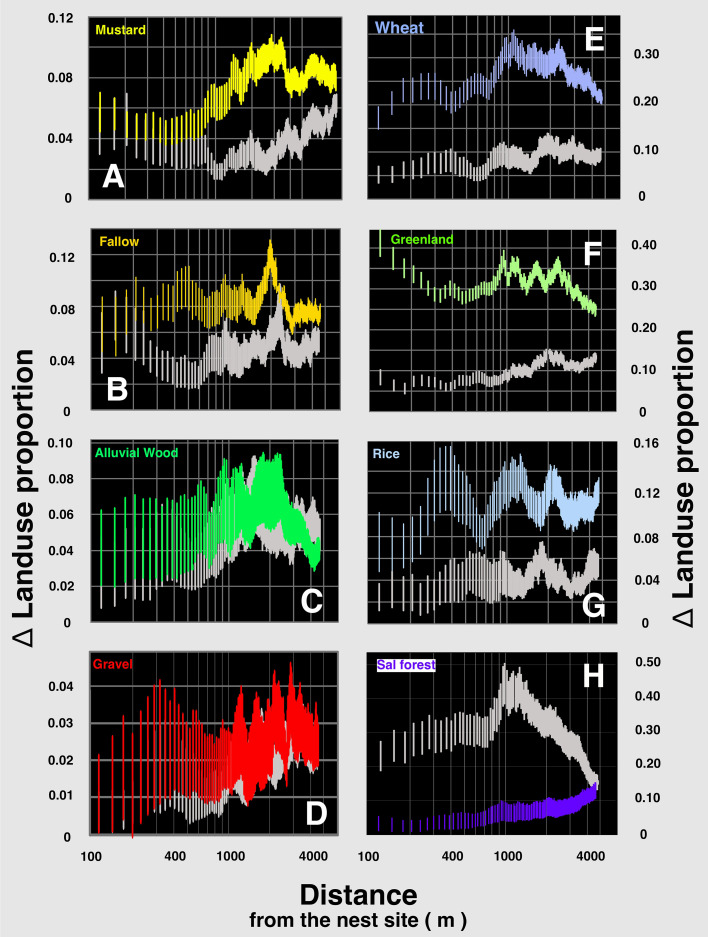
Distance-dependent land-use composition around *Apis dorsata* nesting sites in December 1999. Spatial distribution of major land-use categories surrounding giant honeybee nesting sites in the central Chitwan plains. For each land-use type, mean proportional cover is plotted against distance from nesting sites, with colored vertical bars indicating ± SE and the mean located at the midpoint of each bar. These profiles thus depict distance-dependent land-use composition from the colonies’ perspective (“bee-eye”), integrating information from approximately 300 colonies across 55 nesting sites. Grey vertical error bars represent the corresponding expectation from 55 randomly placed reference points. Panels show distance profiles for mustard **(A)**, fallow land **(B)**, alluvial forest **(C)**, gravel/infrastructure **(D)**, wheat **(E)**, grassland **(F)**, rice **(G)**, and Sal forest **(H)**. Distances are measured radially from nesting sites up to 4.5 km. Deviations between colored and grey profiles indicate systematic over- or under-representation of specific land-use types around nesting sites relative to random locations.

**Fig 7:**
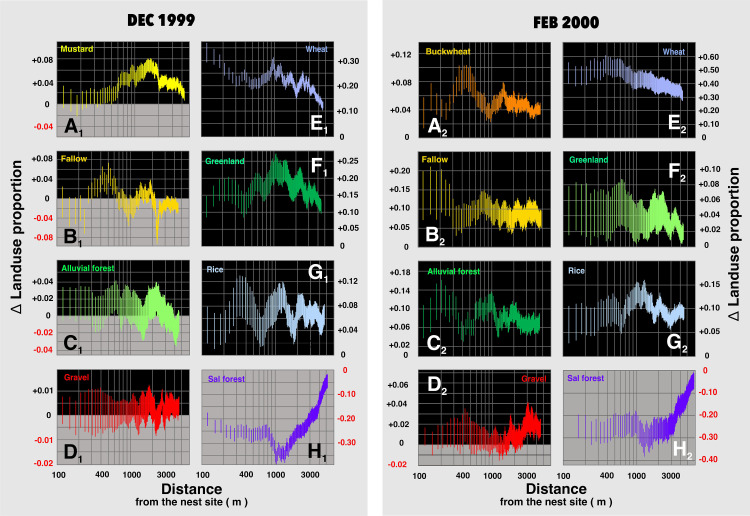
Distance-dependent differences between nesting sites and random locations in land-use composition in December 1999 and February 2000. Radial profiles illustrate how land-use composition surrounding nesting sites differs from randomly selected locations in the central Chitwan plains. For each land-use category, the differential mean proportional cover (±SE) is shown as a function of distance from nesting sites. The difference is calculated as the mean proportional cover around nesting sites (“bee-eye” perspective; ≈ 300 colonies across 55 nesting sites) minus the corresponding mean proportional cover around 55 randomly placed reference points (as shown in Fig. 6). Thus, positive values indicate over-representation of a land-use type around nesting sites relative to random locations, whereas negative values indicate under-representation. Panels A₁–H₁ correspond to December 1999 (mustard-flowering season), and panels A₂–H₂ to February 2000 (buckwheat-flowering season). Land-use categories are: (A) mustard/buckwheat, (B) fallow, (C) alluvial forest, (D) gravel/infrastructure, (E) wheat, (F) dry grass/open green, (G) rice, and **(H)** Sal forest. Distances are shown radially up to 4.5 km from nesting sites.

**Fig 8:**
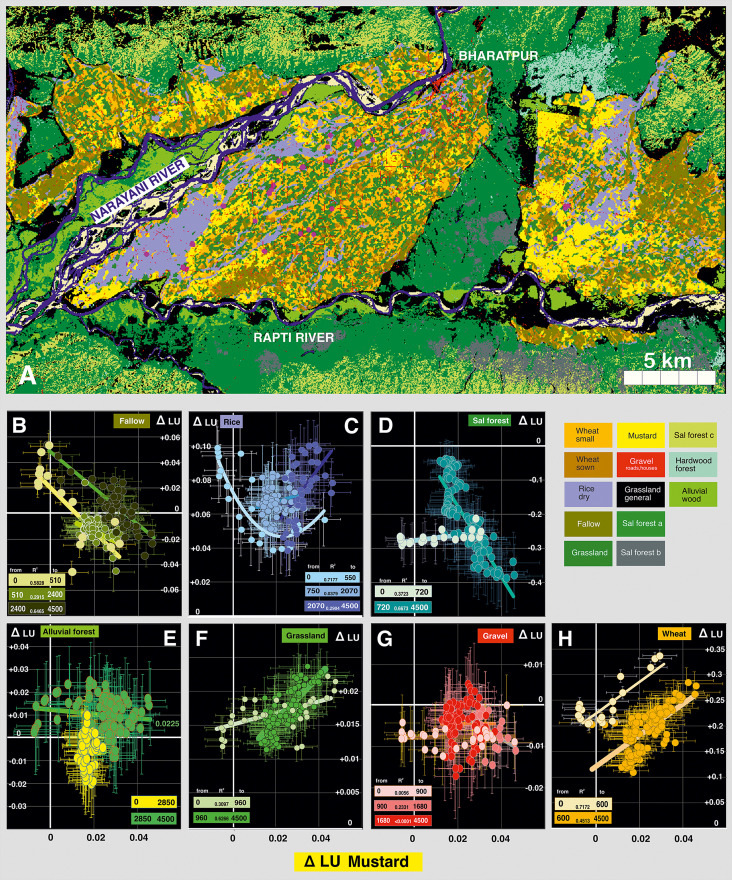
Spatial correlation between mustard and other land-use components (December 1999) derived from LANDSAT imagery. **(A)** Land-use classification of the central Chitwan Valley showing 55 Apis dorsata nesting sites (red dots); pixel size: 15 × 15 **m. (B–H)** Relationships of Δ LU_n-r_ values between mustard (x-axis:) and the respective land-use components (y-axis), where Δ LU_n-r_ denotes the difference in proportional area around nesting sites relative to random sites. Positive values indicate over-representation near nests, negative values under-representation. Linear regressions are fitted for color-coded, predefined distance intervals, indicated by background-colored bars that display both the corresponding “from–to” distance ranges (in meters) and the R² values (coefficient of determination). These relationships illustrate how mustard co-varies spatially with fallow **(B)**, rice **(C)**, Sal forest **(D)**, alluvial forest **(E)**, grassland **(F)**, gravel (roads and houses; **G)**, and wheat (H) within a 4.5 km radius of colonies. Together, the panels reveal complementary associations of mustard with open agricultural habitats (rice, wheat, grassland), weak or neutral relationships with riparian forests, and strong negative associations with Sal forest and fallow land, reflecting landscape structuring by cultivation intensity and crop rotation. Derived from LANDSAT imagery (USGS/NASA; public domain), processed by the authors.

**Fig 9:**
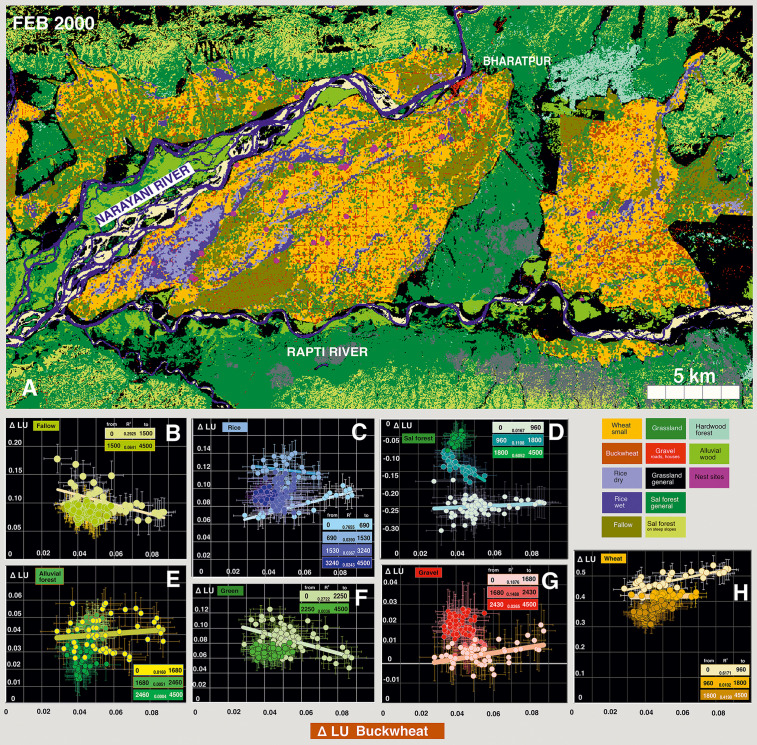
Spatial correlation between buckwheat and other land-use components (February 2000). **(A)** Land-use classification of the central Chitwan Valley showing 55 Apis dorsata nesting sites (magenta) and 55 randomly selected reference sites; pixel size: 15 × 15 **m. (B–H)** Relationships of Δ LU_n-r_ values between buckwheat (x-axis: Δ LU_buckwheat_) and the respective land-use components (y-axis: Δ LU_OTHER_), Definitions of Δ LU_n-r_, distance classes, and regression displays are as in Fig 8. Panels show the spatial co-variation of buckwheat with fallow **(B)**, rice **(C)**, Sal forest **(D)**, alluvial forest **(E)**, grassland **(F)**, gravel (roads and houses; **G)**, and wheat (H) within a 4.5 km radius of colonies. As in Fig 8, open agricultural habitats show positive associations, whereas Sal forest and fallow land show negative associations. Derived from LANDSAT imagery (USGS/NASA; public domain), processed by the authors.

These seasonally shifting, nest-centered landscapes provide the basis for quantitative analyzes of distance-dependent radial land-use profiles (Figs 6, 7) and of spatial co-variation between focal crops and other land-use components (Figs 8, 9). Figs 6 and 7 describe individual land-use profiles, whereas Figs 8 and 9 quantify their relationship to the focal crop across distance.

#### Comparing nest-centered and random landscapes using distance-dependent radial profiles.

Using the December 1999 scene, distance-dependent radial profiles compare land-use composition around the 55 nest-centered landscapes with that around 55 randomly positioned reference landscapes (colored versus grey line distributions in Fig 6). Across distances, these profiles reveal systematic deviations from random spatial expectation, indicating that land-use composition around nesting sites is non-randomly structured.

Several land-use types – including mustard (Fig 6A), wheat (Fig 6E), rice (Fig 6G), fallow land (Fig 6B), and dry grass (Fig 6F) – are consistently over-represented around nesting sites relative to random locations, particularly within a distance range of approximately 1–3 km. This spatial window corresponds closely to the main foraging radius of colonies, suggesting that nesting sites are preferentially embedded within open, cultivation-dominated mosaics rather than within closed or homogeneous land covers. In contrast, Sal forest (Fig 6H) shows the opposite pattern: its proportional cover is consistently lower around nesting sites than around random reference points, with the strongest deficit occurring within the same mid-distance range.

These distance-dependent patterns are synthesized as nest–random land-use contrasts (ΔLU_n–r_) in Fig 7 for the two periods in which colonies were present in Chitwan and crop-derived resources were available (December 1999 and February 2000). The resulting difference curves indicate modest but spatially coherent enrichment of open agricultural and semi-open habitats, whereas the negative Sal-forest contrast mainly reflects the geographical separation between nesting areas in the plains and Sal-forest distributions.

Beyond approximately 3–4 km, land-use proportions around the 55 nest sites converge with those around the 55 randomly selected reference positions, indicating that landscape differentiation between nesting sites and random locations is restricted to the near- and mid-range environment. This convergence is partly inherent to the radial sampling design, because increasing radius progressively incorporates more of the regional landscape matrix, thereby diluting local nest associated deviations from the random reference.

Together, the explicit comparison (Fig 6) and the nest–random contrast analysis term (ΔLU_n–r_; Fig 7) indicate that landscape composition around reproductive nesting sites in December deviates consistently from that expected under random spatial placement. Rather than mirroring the background landscape matrix, nesting sites are embedded in heterogeneous, open, agriculture-dominated settings, whereas the negative Sal-forest contrast largely reflects the separation between nesting areas in the plains and the distribution of continuous Sal forest. Because nesting sites were established before the winter crops reached peak flowering, these patterns likely reflect long-term site fidelity [7,10] and structural landscape features rather than immediate responses to crop availability.

The central question for evaluating nesting-site placement between November and March is whether colonies establish nests within landscapes that subsequently offer predictable access to high-value forage, given that nest placement occurs before mustard flowering and remains fixed until migration. At first sight, across all analyzed land-use components, only two distance–magnitude relationships in Fig 7 exhibit pronounced, well-defined maxima: mustard (Fig 7A_1_) in December and buckwheat (Fig 7A_2_) in February. These peaks indicate that, in each period, a single mass-flowering crop dominates the foraging landscape experienced by colonies in central Chitwan, but at distinct spatial scales: mustard in December 1999 exerts its strongest influence at intermediate distances (≈600–2000 m), whereas buckwheat in February 2000 is concentrated closer to nesting sites (≈200–1000 m).

#### Co-occurrence and trade-offs between focal crops and surrounding land use.

In December 1999, mustard was the dominant focal (forage-relevant) crop and exhibited clear, structured spatial relationships with other land-use components (Fig 8). Strong positive associations were most pronounced with wheat (Fig 8H), and to a lesser extent with rice at larger distances (Fig 8C) and with grassland beyond approximately 950 m (Fig 8F). These associations are strongest at intermediate to larger spatial scales, indicating that these land-use types co-occur within the same agricultural mosaics shaped by farmer management and crop rotation**, r**ather than forming immediately adjacent fields. In this sense, positive association reflects shared placement within broader cultivation zones where mustard is preferentially grown, consistent with coordinated planting decisions rather than fine-scale field-level adjacency.

In contrast, mustard showed pronounced negative associations with Sal forest (Fig 8D) and fallow land (Fig 8B) reflecting spatial segregation between intensively cultivated areas and forested or temporarily uncultivated land. Alluvial forest (Fig 8E) and gravel-dominated areas (Fig 8G) remained close to neutral across distances, indicating a stable background element largely independent of crop distribution.

Taken together, the spatial correlation analyzes for December 1999 and February 2000 (Figs 8, 9) reveal partially parallel correlation structures across seasons: although buckwheat replaced mustard as the focal crop in February, it exhibited a spatial correlation pattern closely mirroring that of mustard in December. Buckwheat showed strong positive associations with wheat (Fig 9H), rice (Fig 9C), indicating that both crops occupy the same intensively cultivated, sun-exposed agricultural matrix. As in December, fallow land (Fig 9B) and grassland (Fig 9F) showed negative associations with nesting sites characterized by high buckwheat representation, consistent with seasonal turnover of cultivated fields. Sal forest (Fig 9D) also showed a negative association, although this likely reflects its limited occurrence in the plains rather than a specific nesting response. Alluvial forest (Fig 9E) and gravel (Fig 9G) again showed no systematic relationship with the focal crop, indicating their role as relatively stable background components of the landscape.

### Crop-to-crop rotation between December 1999 and February 2000

**Fig 10:**
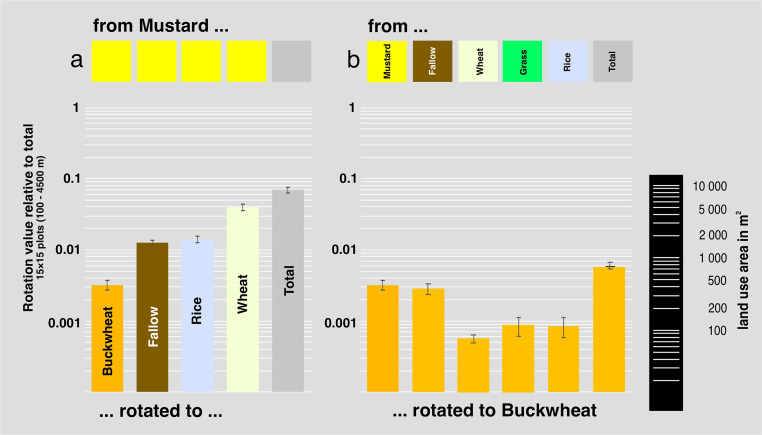
Crop-to-crop rotation around *A. dorsata* nesting sites between December 1999 and February 2000. Rotation values were calculated from 15 × 15 m land-use plots within a 100–4,500 m radius around each nest site using concentric radial distance bands; colored bars show mean proportions ± SE (small black lines). **(a)** Transitions of plots classified as mustard in December 1999 into other land-use categories by February 2000. Colored bars represent the proportions of plots originally occupied by mustard that transitioned into buckwheat, fallow, rice, or wheat. The summed contribution across all categories (“Total”, in grey color) corresponds to the full crop area per nest-centered landscape, shown separately for mustard in December. Within a 4 500 m radius around a nest site, mustard occupied on average 8,750 ± 1,111 m² (means ± SE). Of this area, 418 ± 75 m² (4.78%) was converted to buckwheat by February. **(b)** Converse rotation pattern showing the proportion of plots in each December 1999 land-use category that transitioned into buckwheat by February 2000. This perspective highlights which crops most frequently precede buckwheat in the seasonal rotation sequence. From the February perspective, former mustard fields accounted for nearly half (47.82%) of the total buckwheat area (see “total” bar in grey). The black–white logarithmic scale bar indicates the corresponding land-use area scaled in m².

Crop-to-crop rotation analysis demonstrates substantial seasonal turnover of cultivated land within the foraging landscapes surrounding *Apis dorsata* nesting sites (Fig 10). Using nest-centered circular rotation analysis across radial distance bands (100−4,500 m; see Fig 3), we quantified how land-use categories mapped in December 1999 transitioned into different crop types by February 2000 (Fig 10a), and conversely, which December land-use classes contributed to buckwheat cultivation in February (Fig 10b).

To directly quantify crop-to-crop rotation within nest-centered foraging landscapes, Fig 10a tracks seasonal transitions among land-use classes between December 1999 and February 2000. From the December perspective, the fraction of the landscape classified as mustard was reassigned to other land-use categories by February. Within a 4,500 m radius around a nest site, mustard occupied on average 8,750 ± 1,111 m², of which 418 ± 75 m² (4.78%) was converted to buckwheat by February (see scale in m^2^ at the right side of the plot). Larger proportions transitioned to wheat (57.25%), rice (19.71%), and fallow land (18.25%). When expressed relative to the entire circular landscape surrounding a single nest site, the combined rotation value indicates that the vast majority of former mustard fields remained within the agricultural land-use system, with only a minor fraction temporarily left fallow during the seasonal transition. Notably, when expressed relative to the entire 4,500 m landscape surrounding a nest site, the mean area classified as mustard accounts for only 0.0138%, despite appearing much more extensive when viewed qualitatively in the landscape imagery.

The complementary analysis (Fig 10b), tracing February buckwheat back to its December land-use classes, shows that buckwheat fields originated disproportionately from areas previously cultivated with mustard. On average, buckwheat covered 874 ± 114 m² within a single nest-centered landscape, corresponding only to nearly one tenth of the mustard area recorded in December. Almost half of this buckwheat area (47.82%) was converted directly from former mustard fields, while additional contributions originated from wheat (17.05%), rice (8.61%), dry grassland (16.31%), and fallow land (10.20%).

Together, these patterns reveal strong temporal coupling between the two dominant mass-flowering crops of the season: approximately half of the buckwheat fields present in February originate from land previously occupied by mustard in December, indicating directed seasonal crop succession within the agricultural matrix surrounding *Apis dorsata* nesting sites. Rather than representing random crop turnover, this rotation reflects a structured replacement of one dominant floral resource by another. This continuity offers one plausible explanation for the link between the December and February landscape configurations analyzed elsewhere and helps explain why mustard and buckwheat function as temporally alternating, yet spatially conserved, focal crops within the foraging environment of Apis dorsata colonies.

### Diagnostic strength and spatial precision of land-use signals around *Apis dorsata* nesting sites

**Fig 11:**
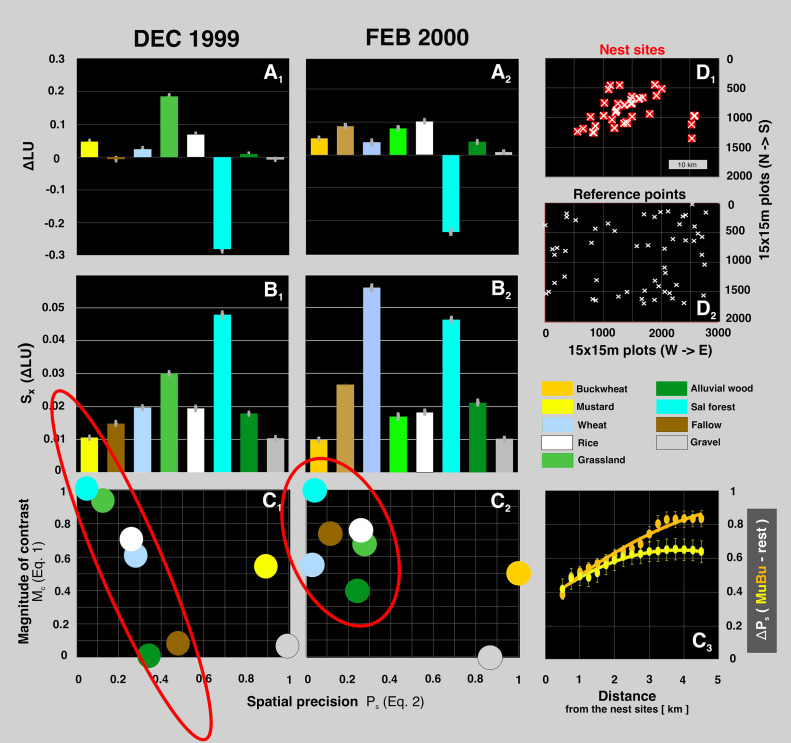
Overview of nest–random land-use contrasts, their variability, and the relationship between signal strength and spatial precision in December 1999 and February 2000 within the biologically relevant foraging range (500–4500 m). Data are shown for December 1999 (left column) and February 2000 (middle column). (A₁, A₂) Mean nest–random land-use contrasts (ΔLUₙ₋_ᵣ_ ± SE) across nesting sites. Positive values indicate enrichment around nest sites, whereas negative values indicate depletion relative to random reference locations. (B₁, B₂) Spatial variability of the nest–random contrasts, expressed as the standard deviation among nesting sites (mean Sₓ [ΔLUₙ₋_ᵣ_] ± SE); lower values indicate higher spatial consistency across colonies. (C₁, C₂) Joint representation of contrast magnitude (ordinate: M_c_; Eq. 1, see Methods) and spatial precision (abscissa: P_s_; Eq. 2, see Methods), calculated for the maximum nest-centred distance of 4,500 m, as also shown in panel C₃. Each point represents a land-use category, color-coded according to the scale at right (mustard, buckwheat, wheat, rice, grassland, fallow, Sal forest, alluvial forest, gravel). Red envelopes indicate Mahalanobis confidence regions, defining the empirically feasible range of relationships between contrast magnitude and spatial precision imposed by landscape structure and sampling design. The positions of mustard (December) and buckwheat (February) fall outside the 95% Mahalanobis confidence regions (corresponding to P < 0.05), indicating that both mass-flowering crops combine moderate contrast strength with unusually high spatial precision. Gravel (grey) serves as a reference category (see S5 k2 Fig), exhibiting minimal contrast magnitude and negligible spatial variability, consistent with its limited relevance in nest-centered landscapes. (C₃) Distance-dependent profiles of spatial precision (P_s_), comparing the focal mass-flowering crops—mustard in December and buckwheat in February—with the remaining land-use classes (means ± SE across 55 nesting sites). Regression curves (R² > 0.90) illustrate how crop-specific contrasts emerge and intensify with increasing distance from nests, whereas the remaining land-use categories remain within the Mahalanobis confidence region. (D₁, D₂) Spatial distribution of nest sites (D₁) and randomly selected reference locations (D₂) within the study area, shown as 15 × 15 m plot positions underlying the nest–random comparisons. Derived from LANDSAT imagery (USGS/NASA; public domain) and processed by the authors.

Fig 11 provides an integrated overview of how land-use components around the 55 Apis dorsata nesting sites differ from those around 55 randomly placed reference locations. Within the biologically relevant foraging range (< 3000 m), two complementary properties of the nest–random contrast are considered: its magnitude and its spatial consistency among nesting sites. These were combined into the derived metrics contrast strength (M_c_; Eq. 1) and spatial precision (P_s_; Eq. 2), allowing land-use components with strong but site-specific deviations from random expectation to be distinguished from those showing weaker yet spatially consistent patterns across colonies. Plotted jointly in a common signal–precision space, the two metrics identify land-use components that are both strongly and consistently associated with nesting sites, thereby separating robust landscape signals from context-dependent or incidental patterns. Estimates of signal precision are therefore conditional on the spatial definition of the reference landscapes.

Across both seasons (Figs 11A_1_–11C_1_: December 1999; Figs 11A_2_–11C_2_: February 2000), land-use categories differ markedly in how strongly and how consistently they are associated with nesting sites. Open agricultural and semi-open habitats generally show positive contrasts, whereas Sal forest exhibits consistently negative contrasts (Figs 11A_1_,11A_2_), a pattern largely attributable to its limited occurrence in the plains.

However, the synoptic representation reveals that not all positive contrasts are equivalent. Widespread crops such as wheat and rice show moderate enrichment around nesting sites, but their contrasts are comparatively diffuse and spatially variable, as reflected by higher Sx [Δ LUn–r] values (Figs 11B_1_, 11B_2_) and weaker positioning in the precision–strength space (Figs 11C_1_, 11C_2_).

In contrast, the seasonally dominant mass-flowering crops – mustard in December 1999 and buckwheat in February 2000 – stand out as exceptional signals. Although their absolute land-use coverage (Figs 11A_1_, 11A_2_) is smaller than that of wheat or rice, their nest–random contrasts are both strong and spatially coherent, placing them close to the right boundary of the empirical precision–strength field (Figs 11C_1_, 11C_2_). Factually, this signal–precision analysis separates statistically mustard in December and buckwheat in February from the bulk of other land-use components. Their positions outside the central Mahalanobis confidence regions (red envelopes in Figs 11C₁,11C_₂_; P < 0.05) indicate that both mass-flowering crops combine moderate contrast strength with unusually high spatial precision. This distinguishes them from other land-use components that may show strong nest–random contrasts but substantially lower spatial consistency across nesting sites.

## Discussion

### Apparent anticipatory nest-site placement under predictable landscape dynamics

In the Chitwan plains, *Apis dorsata* colonies establish seasonal nesting sites each November, well before mustard flowering begins [15,27,29,30]. Any favourable foraging outcomes later in the season must therefore originate from decisions made prior to the emergence of dominant floral resources, implying that site selection reflects predictable landscape dynamics rather than immediate forage availability. Once established, nesting locations may be maintained through population-level site fidelity or the repeated use of traditional nesting areas across years [7,10]. Such long-term spatial continuity is most plausibly mediated at the colony level by queens, whose lifespan spans multiple seasons, in contrast to the worker population, which undergoes rapid turnover within a single nesting cycle (see, e.g., [7,10,24,31,32]). Together, these constraints emphasise the importance of persistent, seasonally recurring spatial signals in the agricultural matrix for understanding nest-site placement in this species.

### A colony-centred diagnostic framework reveals scale-specific land-use signals

Using a nestsite-centred, distance-resolved analytical framework derived from satellite imagery (S4 Fig; Figs 4–9), we quantified how land-use components are spatially configured around nesting sites relative to random expectation. This framework isolates nest–random land-use contrasts (ΔLU_n–r_) and evaluates them by both magnitude and spatial consistency, thereby identifying components whose representation around nests differs systematically from the surrounding landscape.

In both December 1999 and February 2000 (Fig 7), pronounced peak-shaped distance–magnitude relationships within the biologically relevant foraging range (approximately 500–2,000 m) were observed exclusively for mustard and buckwheat, two crops of direct nutritional relevance to honeybees. No other land-use components resolved in the satellite imagery exhibited comparable scale-specific maxima. At larger distances, nest-associated signals converged towards random reference landscapes, indicating that the strongest differentiation is restricted to the near- and mid-range foraging environment. Local ecological factors, including environmental barriers, colony density, and inter-colony competition, may nevertheless modulate the precise distance at which convergence occurs.

The peak-shaped patterns of mustard and buckwheat are statistically significant and, critically, reproducible across nesting sites and seasons, making them robust empirical reference patterns. These qualitative signals are reinforced by the signal–precision analysis: mustard in December and buckwheat in February are separated (P < 0.05) from most other land-use components and occupy regions of moderate contrast strength and high spatial precision (Figs 11C_1_,11C_2_). Their position outside the central Mahalanobis confidence region distinguishes them from components that may exhibit strong contrasts but do so less consistently across nesting sites. At this stage, the signal–precision framework is diagnostic rather than explanatory, identifying consistent spatial differentiation without invoking behavioural preference or causal mechanisms.

### Rotational coupling links seasonal forage signals

Crop-transition analyses (Fig 10) show that mustard and buckwheat are linked by rotational dynamics, with individual plots frequently shifting from mustard in December to buckwheat in February. The associated spatial signals are therefore best interpreted as seasonally alternating expressions of a coherent agricultural structure surrounding nesting sites. Together, these results indicate that mustard and buckwheat are structurally central and seasonally predictable components of the *A. dorsata* foraging landscape. Although this study does not infer active habitat choice or behavioural decision-making, nestsite-centred landscapes are organised in a manner consistent with nesting under predictable crop-rotation regimes.

Mustard and buckwheat are not only conspicuous seasonal land-use classes in the Chitwan plains, but also represent major functional forage resources for *Apis dorsata* during the nesting season. As mass-flowering crops, they may provide concentrated supplies of nectar and pollen during biologically critical phases, including colony establishment, comb construction, brood production, and seasonal movement. Moreover, they may form a temporally complementary sequence of flowering crops, potentially sustaining colony development across successive phases of the nesting season. Nectar supplies carbohydrates for flight and colony energetics, whereas pollen provides proteins and other nutrients required for brood development. By contrast, dominant cereal crops (rice, wheat, maize) are wind-pollinated and contribute little direct forage for honeybees. The observed seasonal turnover from mustard to buckwheat is therefore ecologically consistent with the physiological demands of large *A. dorsata* colonies.

Land-use presence, however, does not translate directly into realised resource availability. In this analysis, crop distribution serves as a proxy for the spatial arrangement of potential forage resources, and biological relevance may depend on flowering phenology, floral reward density, and nutrient composition. Accordingly, while the identified patterns are consistent with resource-based nest positioning, they do not resolve the underlying nutritional mechanisms of colony migration or site fidelity; resolving nectar and pollen quality will be essential for a mechanistic account of these processes.

The novelty of this work lies not in documenting seasonal crop turnover per se, but in providing a spatially explicit, null-model–based diagnostic framework [33] that (i) adopts a colony-centred “bee-eye” perspective (Fig 3), (ii) disentangles nest-specific spatial signals from general land-use dynamics (Fig 6), and (iii) quantifies the strength and consistency with which individual land-use components differentiate nestsite-centred landscapes (Fig 11). This approach reveals how large-scale agricultural dynamics translate into biologically relevant spatial signals at the colony level.

### Distinct spatial signals of forage-relevant crops: evaluating alternative explanations

The pronounced separation revealed by the diagnostic framework highlights land-use components of direct nutritional relevance for *Apis dorsata*, prompting the question of which mechanisms could underlie this non-random pattern. Any such explanation must be compatible with the observed spatial precision, seasonal alternation, and nestsite-centred organisation of the data.

A first possibility is that the observed separation reflects a season-specific artefact tied to the cropping sequence of 1999/2000. However, this interpretation is not supported by the spatial and analytical structure of the dataset. The analysis integrates a large and spatially extensive sample (55 nesting sites across >1,200 km²), yielding substantial statistical power. More importantly, the nest–random contrast approach explicitly filters out local variation, retaining only patterns that are consistent across many nesting sites and therefore representative of landscape-scale structure [34–36]. Comparable results emerge in two independent seasonal snapshots (December 1999 and February 2000), despite shifts in the nutritionally dominant crop and its spatial distribution. This apparent discontinuity is bridged by crop rotation, with a substantial fraction of February buckwheat fields derived from former mustard plots (Fig 10). Together, these observations indicate that the separation of nutritionally relevant land-use components reflects system-level agricultural structure rather than transient local effects.

A second concern is that the Chitwan plain may represent a geographically idiosyncratic system. However, despite its local specificity, it exemplifies structural features common across much of the range of *A. dorsata*, including strong seasonal crop rotation, reliance on a limited number of mass-flowering crops, and sharp transitions between intensive agriculture and forest [4,7,32]. Because the analysis is based on relative spatial contrasts rather than absolute crop identity, the observed dominance of a single, spatially precise forage signal is consistent with patterns reported from other agricultural systems characterised by temporally concentrated floral pulses [37–39]. The results therefore most parsimoniously reflect a general landscape mechanism rather than a geographically exceptional case.

A further alternative is that the observed separation arises purely from structural landscape properties, such as openness or accessibility. This explanation is not supported, as structurally similar land-use types show markedly different contrast strengths and levels of spatial precision. Only mustard and buckwheat combine distance-specific peaks with a distinct position in signal–precision space, whereas other open land-use components cluster near the bulk distribution (Fig. 11). Similar decoupling of structural and ecological relevance has been reported in other landscape contexts [39,40].

### Interpretative limits, biogeographic constraints, and falsifiable extensions

This analysis is based on data from 1999–2000 and therefore represents a historical snapshot of colony–landscape relationships. Nevertheless, the identified patterns—particularly the scale-specific structure of signal precision—are likely to reflect underlying behavioural and ecological mechanisms that are more stable than specific land-use configurations. Ongoing fieldwork and re-analysis using contemporary high-resolution satellite data will allow assessment of their persistence under current conditions.

The observed separation of food-relevant and non-relevant land-use signals does not imply that individual foraging workers actively evaluate landscape-scale crop distributions, nor that nest placement reflects individual-level optimisation. Rather, the pattern is consistent with constraints imposed by nesting-site placement within a spatially structured and predictably rotating agricultural landscape.

In the Chitwan plains, suitable nesting substrates are limited: large trees are scarce, while anthropogenic structures increasingly provide attachment sites (S1 Fig). Under such conditions, nesting locations may persist at the colony level across years, reinforcing spatial continuity independently of short-term foraging conditions [7,10]. Comparable decoupling between nest placement and immediate resource distribution is well documented in honeybees more generally [4,41,42]. Throughout, terms such as “diagnostic landscape signals” are therefore used strictly in a statistical sense, following established distinctions between habitat diagnostics and behavioural inference [43–45].

Interpretation is further bounded by spatial and biogeographic constraints. The analysis is restricted to the ~ 40 × 30 km area covered by the available satellite imagery; extending the approach to adjacent regions would provide a direct test of spatial generality. Northward extrapolation is limited by species turnover and geographic separation between lowland *A. dorsata a*nd montane *A. laboriosa* populations, whereas eastern regions exhibit partial sympatry [1,6]. Consequently, patterns identified in Chitwan cannot be directly transferred to high-altitude systems. Qualitative observations from adjacent lowland habitats nevertheless suggest consistency in seasonal aggregation and migration patterns.

Finally, the risk of circular inference is addressed by explicitly comparing nestsite-centred landscapes with randomly placed reference landscapes under identical spatial constraints. This null-model approach tests whether nest surroundings differ systematically from expectations within the same landscape matrix [33,46]. At larger spatial scales, both nest-centred and reference landscapes increasingly integrate regional structure, and local contrasts are therefore attenuated. The convergence of nest-associated signals with random landscapes at greater distances should thus not be interpreted as a strict foraging boundary, but as the outcome of spatial averaging, biological foraging scale, and broader landscape structure, potentially modulated by overlap among neighbouring colonies.

Future analyses should evaluate whether additional land-use elements, such as expanded banana cultivation, contribute to forage availability outside the mustard and buckwheat flowering periods. Together, these considerations delimit interpretation and define falsifiable extensions: recurrence of the pattern across regions, years, or comparable systems would support the proposed mechanism, whereas its absence would call it into question.

## Supporting information

S1 Fig Survey of the Chitwan plains showing nest sites of Apis dorsata. Representative examples of mass nesting at individual sites. (a) The unique “bee house” in the southern part of the plain with nine active colonies nesting on the roadside façade (a₁) during our survey in February 2000. At a subsequent visit in February 2004 (a₂), the roof had been structurally modified to add additional storeys; following this reconstruction, colonies no longer occupied this previously “traditional” nesting site. (b) The main water tower on the Rampur campus, supporting more than fifty active colonies in February 2010. Photo credit: Gerald Kastberger. Published under CC BY 4.0.
(JPG)

S2 Fig Spatial distribution of Apis dorsata nest sites in the central Chitwan plain. Background derived from LANDSAT imagery (USGS/NASA; public domain), processed by the authors, shows the agricultural lowland bounded by surrounding forested foothills. Blue lines indicate major river systems, including the Narayani River flowing from north to southwest and the Rapti River forming the northern boundary of Chitwan National Park. The white rectangle delineates the spatial extent of the study area used for nestsite-centred and random-centred landscape analyses. Pink circles mark the 55 documented nesting sites of *Apis dorsata*.
(JPG)

S3 Fig Foraging Apis dorsata worker bees in the Chitwan plain near Sauraha (February 2010). (a) Collecting nectar from common buckwheat (*Fagopyrum esculentum* Moench), a widely cultivated winter crop in Nepal and a major nectar and pollen source during its flowering period (typically January–February). (b) A forager bee approaching Indian mustard (*Brassica juncea*), the dominant mustard crop in the Chitwan plain, to collect nectar and pollen; an important early-season floral resource for *Apis dorsata*. Photo credit: Gerald Kastberger. Published under CC BY 4.0.
(JPG)

S4 Fig Survey over the analytical framework linking spatial data to biological inference. Satellite-derived land-use maps of the Chitwan plain provide the basis for all analyzes. For each of 55 nest sites (~300 colonies), colony-centered (“bee-eye”) landscapes were defined using concentric rings (from 100 to 4,500 m in 15m steps) to quantify distance-dependent land-use composition (Figs. 3, 4). Identical ring structures were assessed also around 55 randomly selected reference locations (forming “random-centered” landscapes; Fig. 6), allowing nest-specific spatial structure to be evaluated against null expectations. Nestsite-centered landscapes were further characterized using sector-based metrics that resolve both proportional cover and radial extent of land-use classes (Fig. 5). Direct comparison between nest- and random-centered landscapes yielded distance-dependent land-use contrasts (Δ LUₙ_–ᵣ_; Figs. 6–9), isolating nest-associated spatial signals from background seasonal turnover. Crop-to-crop transitions (Fig. 10), particularly mustard (December 1999) to buckwheat (February 2000), link spatial contrasts to agricultural rotation dynamics. Finally, contrast magnitude (M_c_) and spatial precision (P_s_) were integrated for both seasons (Fig. 11) to identify land-use components showing strong and consistent nest-associated signals. Mustard (December) and buckwheat (February) emerge as diagnostically relevant crops. Together, this framework demonstrates how nest-site-centered analyzes and null-model comparisons reveal biologically meaningful landscape structure consistent with an apparently “anticipatory” pattern of nest-site selection, shaped by queen-mediated site fidelity, the reuse of traditional nesting sites recognized by wax remnants, and the availability of suitable attachment structures.
(JPG)

S5 Fig Spatial distribution of land-use components around nest sites across seasons. This figure extends with the panels f-k the analysis presented in Fig. 2a-e. Nest-centered land-use overlays for the central Chitwan plain shown for three survey dates (13 December 1999, 15 February 2000, and 3 April 2000). Columns represent survey dates; rows represent individual land-use components, displayed as color-coded overlays on a common grayscale base image. White rectangles indicate the analyzed landscape window surrounding nest sites. Rows depict the following land-use categories (see color coding within panels): f, fallow; g₁–g₂, Sal forest; h, alluvial forest; i, pinus forest; i, htm-code which is mainly built-up / infrastructure-related land use; k₁–k₂, river channels and gravel bars). The panels illustrate non-forage and structural land-use components (rows f–k) which show comparatively stable spatial distributions across seasons. Fallow land (panels d) increases in spatial extent from December 1999 to April 2000, consistent with the complete harvest of buckwheat and wheat by March 2000. In contrast, Sal forest areas (panels g) exhibit additional spatial patches in April, necessitating the introduction of an additional classification class, likely reflecting seasonal changes in leaf coloration and flowering status. These overlays provide the spatial context for the distance-resolved and nest–random contrast analyzes presented in Figs. 4–9. The inclusion of temporally stable land-use components (e.g., forest, water bodies, and large built structures) across all three survey dates is intentional, as their consistent spatial patterns provide an internal check on the accuracy of land-use classification and increase confidence in the reliability of the crop-related analyzes. Derived from LANDSAT imagery (USGS/NASA; public domain), processed by the authors.
(JPG)
