# Supplementary figures and images for: Nesting in anticipation: Spatial ecology of giant honey bees (Apis dorsata) in relation to crop succession mapped by remote sensing
Source: PLoS One. 2026 Jun 24;21(6):e0347045. doi: 10.1371/journal.pone.0347045 (PMC13293442; doi:10.1371/journal.pone.0347045)

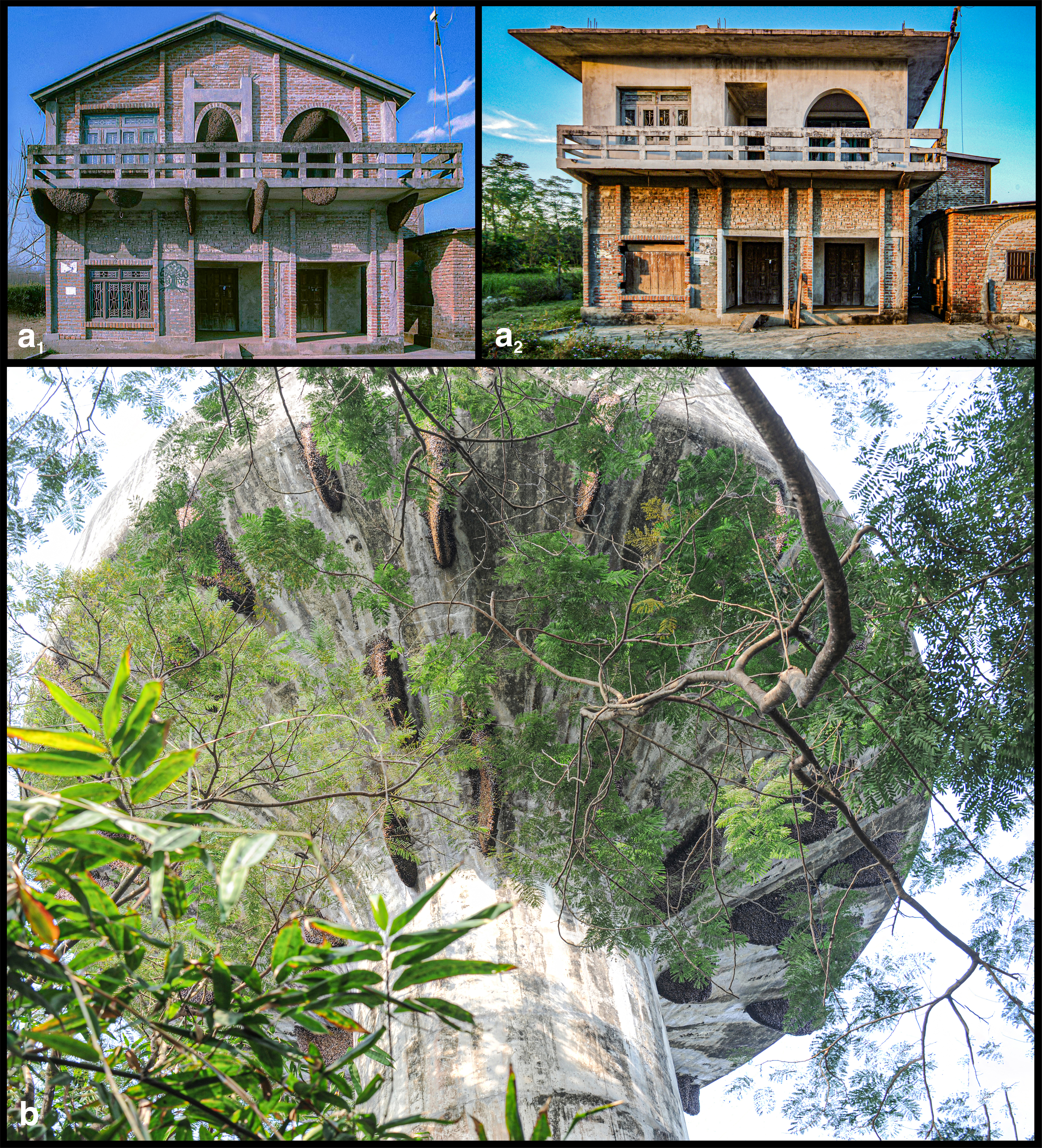

Supplement: S1 Fig — Representative examples of mass nesting at individual sites. (a) The unique “bee house” in the southern part of the plain with nine active colonies nesting on the roadside façade (a₁) during our survey in February 2000. At a subsequent visit in February 2004 (a₂), the roof had been structurally modified to add additional storeys; following this reconstruction, colonies no longer occupied this previously “traditional” nesting site. (b) The main water tower on the Rampur campus, supporting more than fifty active colonies in February 2010. Photo credit: Gerald Kastberger. Published under CC BY 4.0. (JPG) [file pone.0347045.s001.jpg]

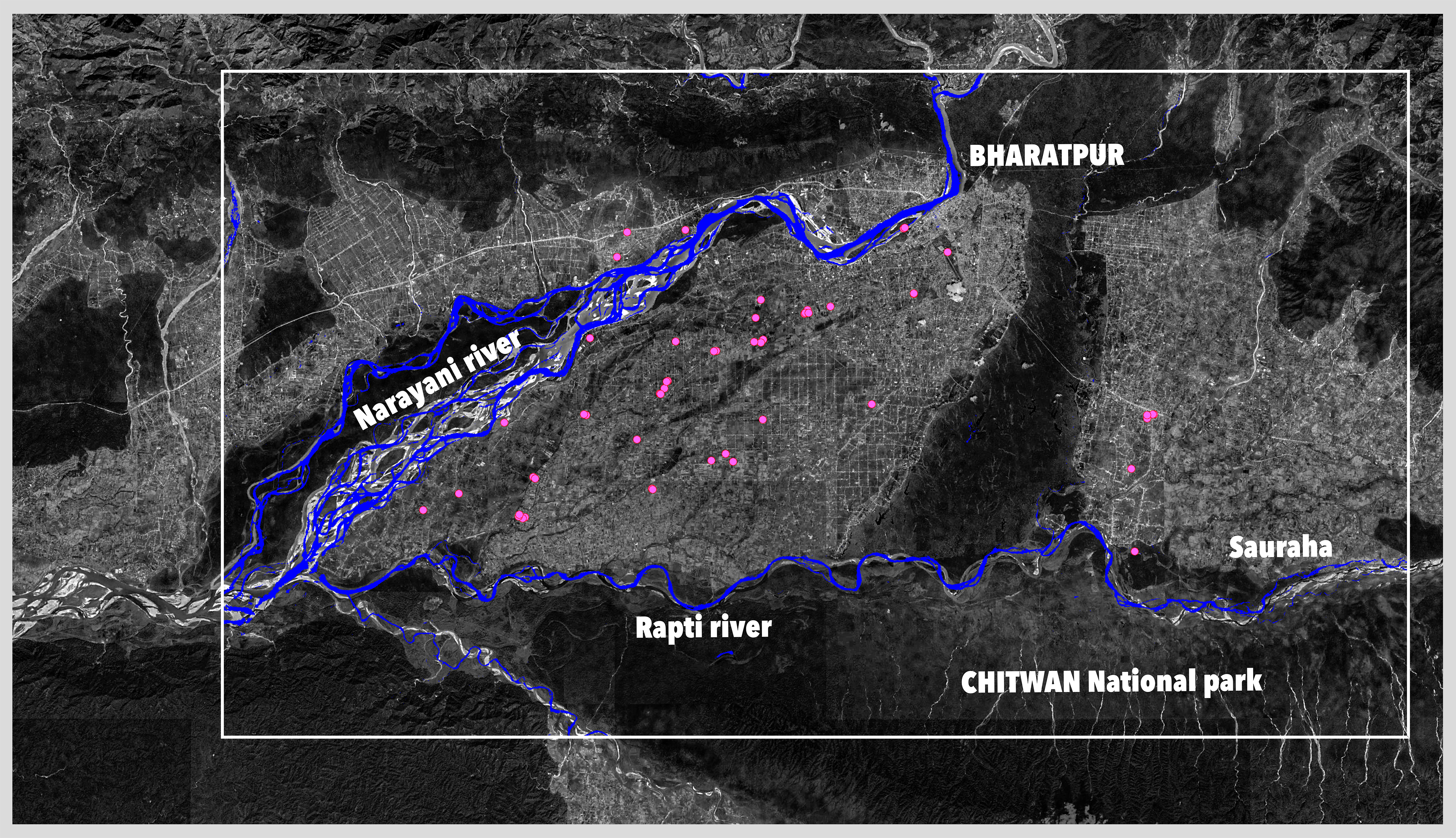

Supplement: S2 Fig — Background derived from LANDSAT imagery (USGS/NASA; public domain), processed by the authors, shows the agricultural lowland bounded by surrounding forested foothills. Blue lines indicate major river systems, including the Narayani River flowing from north to southwest and the Rapti River forming the northern boundary of Chitwan National Park. The white rectangle delineates the spatial extent of the study area used for nestsite-centred and random-centred landscape analyses. Pink circles mark the 55 documented nesting sites of Apis dorsata. (JPG) [file pone.0347045.s002.jpg]

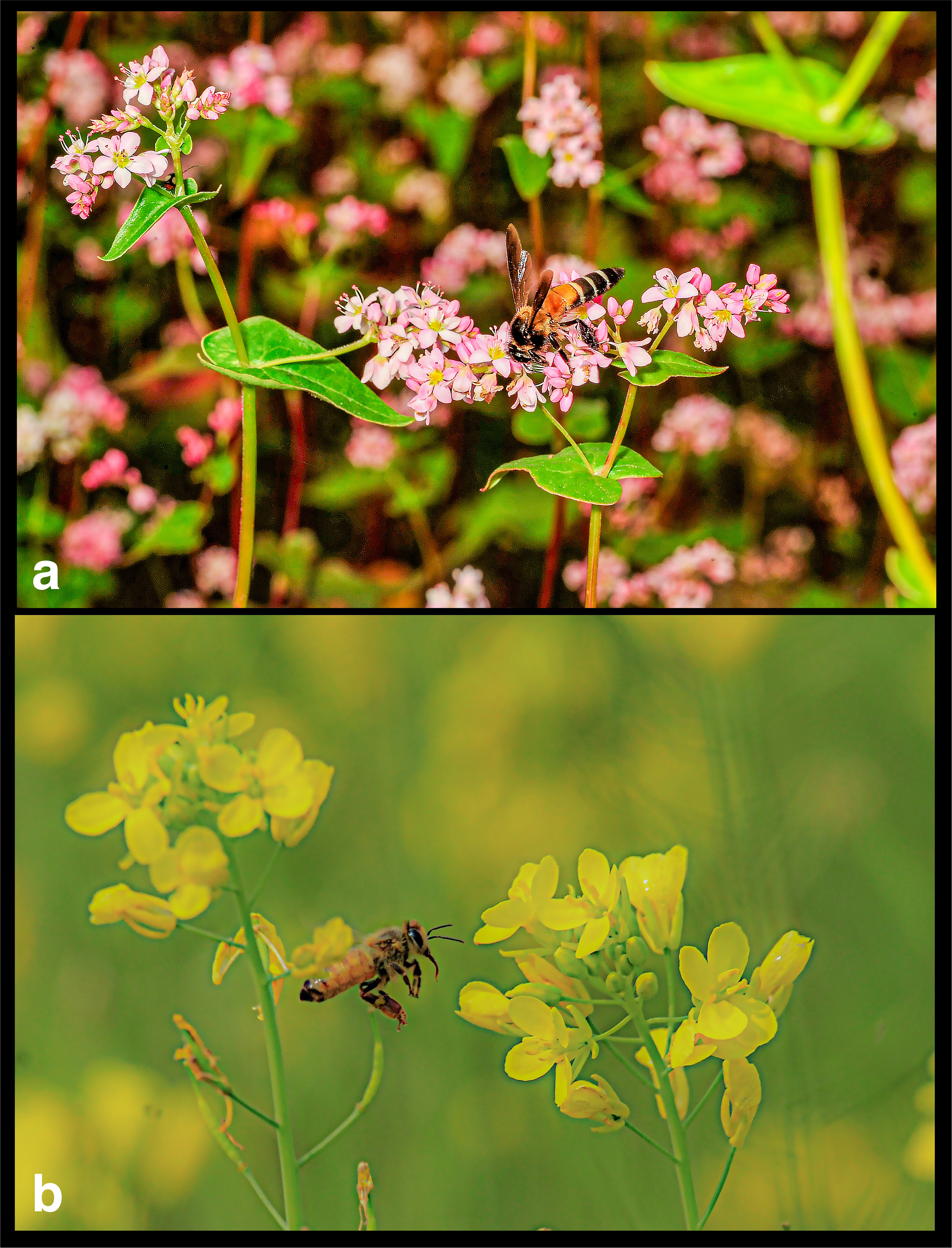

Supplement: S3 Fig — (a) Collecting nectar from common buckwheat (Fagopyrum esculentum Moench), a widely cultivated winter crop in Nepal and a major nectar and pollen source during its flowering period (typically January–February). (b) A forager bee approaching Indian mustard (Brassica juncea), the dominant mustard crop in the Chitwan plain, to collect nectar and pollen; an important early-season floral resource for Apis dorsata. Photo credit: Gerald Kastberger. Published under CC BY 4.0. (JPG) [file pone.0347045.s003.jpg]

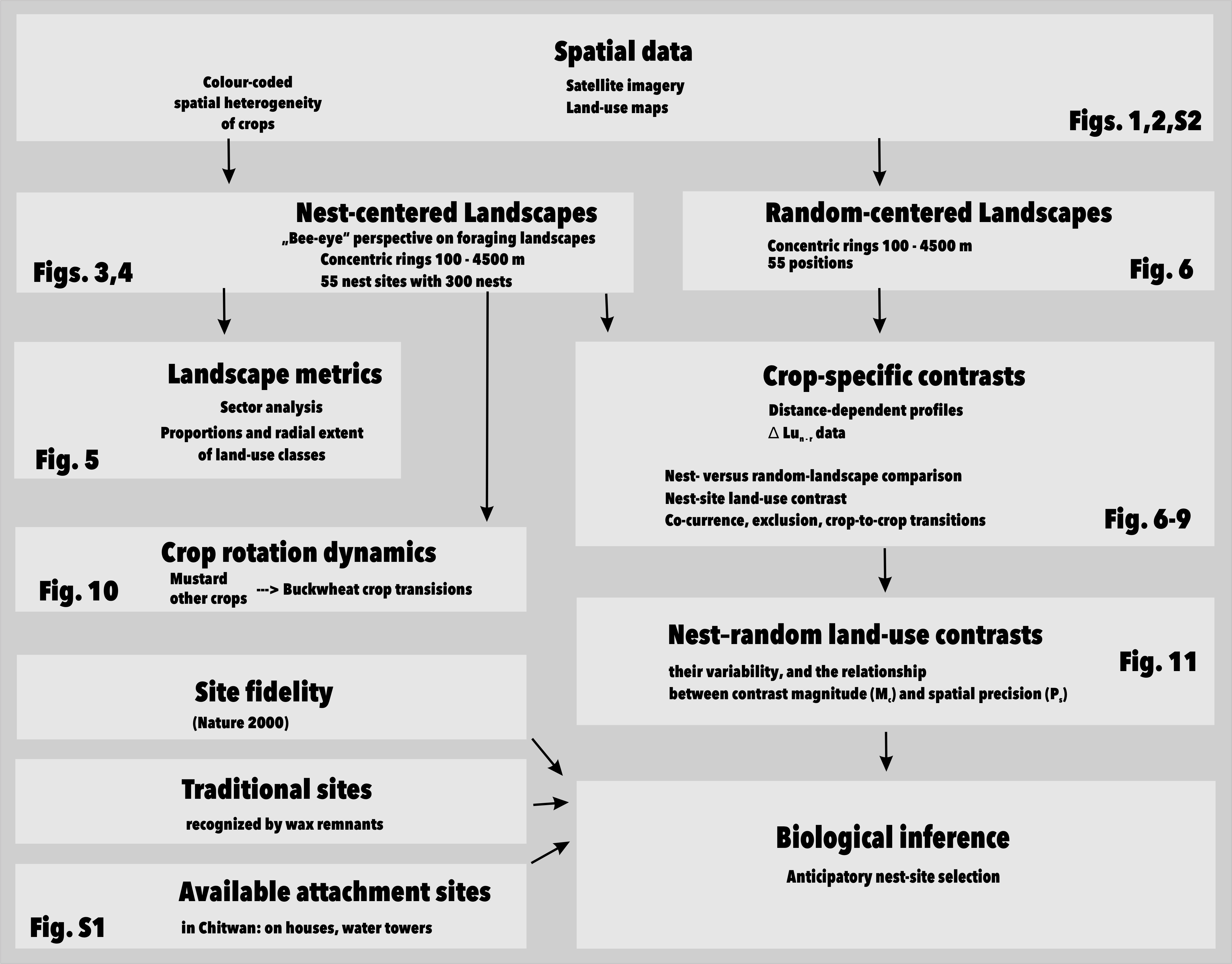

Supplement: S4 Fig — Satellite-derived land-use maps of the Chitwan plain provide the basis for all analyzes. For each of 55 nest sites (~300 colonies), colony-centered (“bee-eye”) landscapes were defined using concentric rings (from 100 to 4,500 m in 15m steps) to quantify distance-dependent land-use composition (Figs. 3, 4). Identical ring structures were assessed also around 55 randomly selected reference locations (forming “random-centered” landscapes; Fig. 6), allowing nest-specific spatial structure to be evaluated against null expectations. Nestsite-centered landscapes were further characterized using sector-based metrics that resolve both proportional cover and radial extent of land-use classes (Fig. 5). Direct comparison between nest- and random-centered landscapes yielded distance-dependent land-use contrasts (Δ LUₙ–ᵣ; Figs. 6–9), isolating nest-associated spatial signals from background seasonal turnover. Crop-to-crop transitions (Fig. 10), particularly mustard (December 1999) to buckwheat (February 2000), link spatial contrasts to agricultural rotation dynamics. Finally, contrast magnitude (Mc) and spatial precision (Ps) were integrated for both seasons (Fig. 11) to identify land-use components showing strong and consistent nest-associated signals. Mustard (December) and buckwheat (February) emerge as diagnostically relevant crops. Together, this framework demonstrates how nest-site-centered analyzes and null-model comparisons reveal biologically meaningful landscape structure consistent with an apparently “anticipatory” pattern of nest-site selection, shaped by queen-mediated site fidelity, the reuse of traditional nesting sites recognized by wax remnants, and the availability of suitable attachment structures. (JPG) [file pone.0347045.s004.jpg]

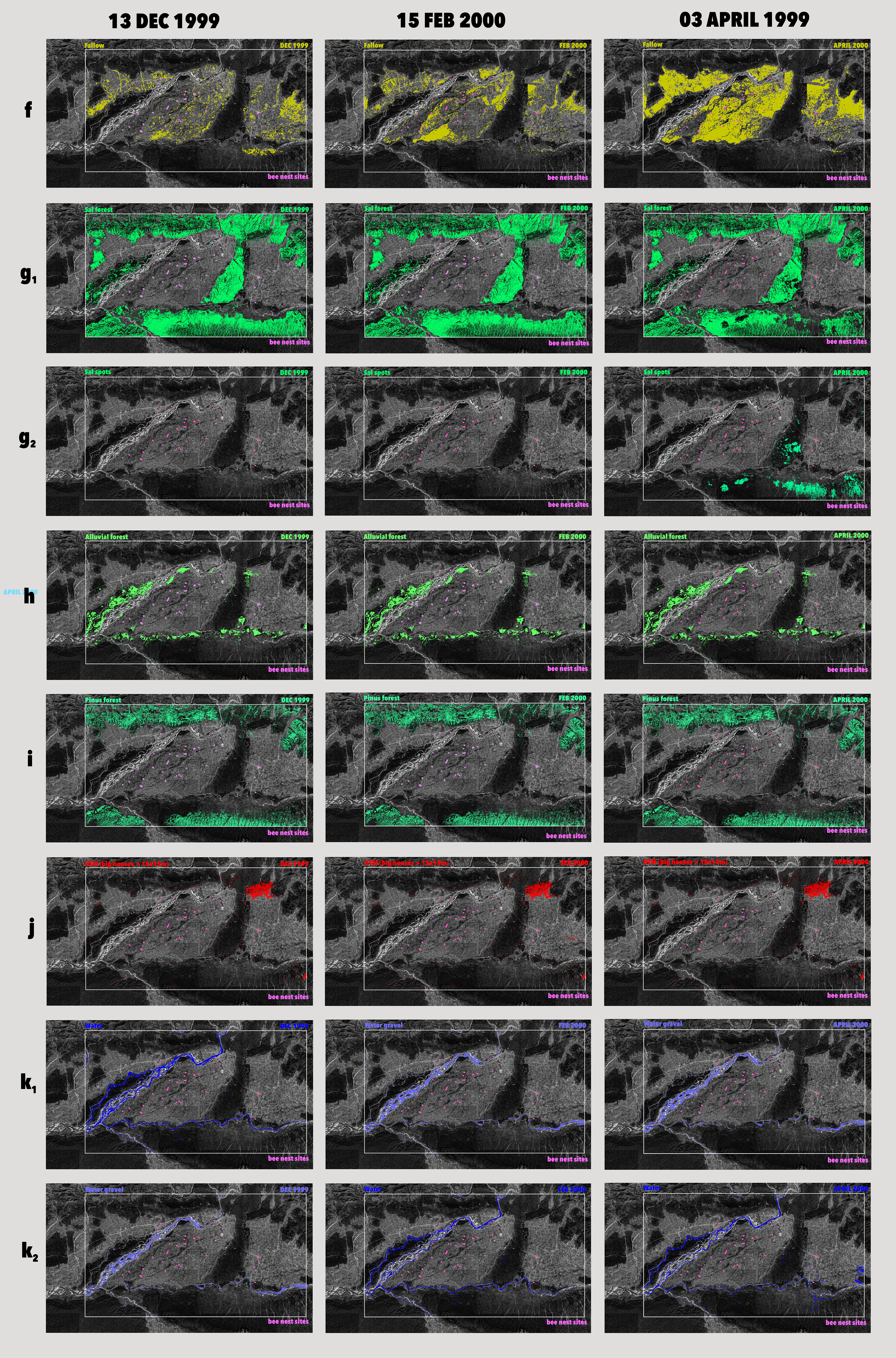

Supplement: S5 Fig — This figure extends with the panels f-k the analysis presented in Fig. 2a-e. Nest-centered land-use overlays for the central Chitwan plain shown for three survey dates (13 December 1999, 15 February 2000, and 3 April 2000). Columns represent survey dates; rows represent individual land-use components, displayed as color-coded overlays on a common grayscale base image. White rectangles indicate the analyzed landscape window surrounding nest sites. Rows depict the following land-use categories (see color coding within panels): f, fallow; g₁–g₂, Sal forest; h, alluvial forest; i, pinus forest; i, htm-code which is mainly built-up / infrastructure-related land use; k₁–k₂, river channels and gravel bars). The panels illustrate non-forage and structural land-use components (rows f–k) which show comparatively stable spatial distributions across seasons. Fallow land (panels d) increases in spatial extent from December 1999 to April 2000, consistent with the complete harvest of buckwheat and wheat by March 2000. In contrast, Sal forest areas (panels g) exhibit additional spatial patches in April, necessitating the introduction of an additional classification class, likely reflecting seasonal changes in leaf coloration and flowering status. These overlays provide the spatial context for the distance-resolved and nest–random contrast analyzes presented in Figs. 4–9. The inclusion of temporally stable land-use components (e.g., forest, water bodies, and large built structures) across all three survey dates is intentional, as their consistent spatial patterns provide an internal check on the accuracy of land-use classification and increase confidence in the reliability of the crop-related analyzes. Derived from LANDSAT imagery (USGS/NASA; public domain), processed by the authors. (JPG) [file pone.0347045.s005.jpg]
