# Supplementary material for: Nesting in anticipation: Spatial ecology of giant honey bees (Apis dorsata) in relation to crop succession mapped by remote sensing
Source: PLoS One. 2026 Jun 24;21(6):e0347045. doi: 10.1371/journal.pone.0347045 (PMC13293442; doi:10.1371/journal.pone.0347045)
Supplement: Attachment — Submitted filename: Rebuttal letter 1abcd - Response to Reviewers.docx [file pone.0347045.s007.docx]

Response to Editor and Reviewers

We thank the Editor for the careful evaluation of our manuscript. Below we respond to the Editor’s comments point by point (red: editor’s queries; green: my comments).

1. …recommend that you deposit your laboratory protocols in protocols.io

My comment: This study did not involve laboratory procedures; therefore, no lab protocols are available.

2. …code sharing for submissions in which author-generated code underpins the findings in the manuscript.

My comment: **Data availability**
For most figures and analytical chapters, the underlying data have been deposited in a form intended to enable independent verification and, to a large extent, recalculation of the reported results. The file naming scheme is designed so that the purpose of each file can be readily inferred from its name. Each dataset is accompanied by README documentation explaining its structure and relevance to the corresponding analyses. Where useful, analysis macros and scripts used in data processing, including Python, Fiji, and VBA code, are also provided as illustrative examples of the evaluation workflow. In a limited number of cases, however, full self-contained deposition is impractical because the datasets are unusually large or rely on complex cross-referencing among multiple Excel worksheets. In such cases, the relevant files and additional technical information are available from the first author upon reasonable request.

I have added the DOI for the Zenodo repository containing the supplementary data associated with each figure. This information is provided after the Acknowledgments, under the heading **Data Availability**:

“All figure-associated datasets, Excel workbooks, analytical macros, Python scripts, and supporting README files underlying this study are available from the Zenodo repository: <https://doi.org/10.5281/zenodo.20157991>”.

3. In your Methods section, please provide additional information regarding the permits you obtained for the work. Please ensure you have included the full name of the authority that approved the field site access and, if no permits were required, a brief statement explaining why.

My comment: **We add this paragraph into Methods**

**Permits and field access**

The study was based on non-invasive field observations and spatial analyses. No animals were collected, handled, or experimentally manipulated. Access to the relevant field sites was obtained with the agreement of landowners / local authorities / local communities / site managers. No specific formal permit was required for this work under the regulations applicable at the time of the study.

4. My comment: The manuscript has been prepared and submitted as a Word document, not as a LaTeX submission. Therefore, the PLOS LaTeX template is not applicable.
5. My comment: We have removed funding-related information from the Acknowledgments section, in accordance with PLOS ONE guidelines.

We request the Funding Statement to be updated as follows:

“This study was supported by the Austrian Science Fund (FWF) project P13210, ‘Migratory Behaviour and Colony Aggregation in Giant Honeybees.’ Publication costs were funded by the University of Graz.”

6. My comment: We have checked and harmonised the funding information across the ‘Funding Information’ and ‘Financial Disclosure’ sections. The entries now match and include the correct grant number (FWF project P13210) as well as publication funding by the University of Graz.
7. My comment: We request that the Funding Statement be updated as follows:
   “This study was supported by the Austrian Science Fund (FWF) project P13210, ‘Migratory Behaviour and Colony Aggregation in Giant Honeybees.’ Publication costs were funded by the University of Graz.”

Role of the funders:
“The funders had no role in study design, data collection and analysis, decision to publish, or preparation of the manuscript.”

8. My comment: We have revised all figures listed by the editor (Figs. 1–5, 8, 9, S2 and S5) to ensure full compliance with PLOS ONE copyright and licensing requirements.

All background imagery is now derived exclusively from LANDSAT data (USGS/NASA; public domain) and has been processed and reclassified by the authors. The figures therefore represent analytical products and do not reproduce any original satellite imagery.

Any previously included third-party imagery (e.g. Google Earth) has been removed.

Accordingly, all figures constitute replacement figures that comply with the CC BY 4.0 license. Figure captions have been updated to include appropriate source attribution.

The analysis is based on LANDSAT source data, which are now publicly available through USGS archives. Equivalent scenes can be accessed using the scene identifiers and acquisition dates provided in the Methods section, ensuring reproducibility and transparency.

9. My comment: Supplementary Figures S1 and S3 are original photographs created by the first author (Gerald Kastberger), who is the copyright holder. These figures are now published under the Creative Commons Attribution License (CC BY 4.0). The figure captions have been updated accordingly.

No additional citation requests requiring action were identified.

10. My comment: We have reviewed the reference list and corrected minor inconsistencies and formatting issues to ensure completeness and accuracy.

My comment: Addendum

- We attach a corrected version of the manuscript in which the corrections are highlighted. Please note that this version does not yet fully reflect the latest status, as we have since completed a final polishing round. The final draft is now shorter than the version attached here.
- All figures have been checked and improved with respect to formatting, including line style, line thickness, and consistent text size.
- The file names have been selected to communicate the content of each figure clearly and concisely.
- Equations 1 and 2 were prepared using the Microsoft Word equation editor. We would prefer to retain this formatting in the manuscript.
- I have cited the Figures according your regulations e.g. “.. whereas the satellite perspective (Fig. 2B; Fig. 4C₁,C₄) reveals a strong association with the Narayani River and its floodplain. Grassland (Fig. 2E; Fig. 4D₂,D₃) and fallow land (S2f Fig; Fig. 4E₂,E₃)..”

We thank both reviewers for their constructive comments. Below, we respond to each point in detail. For clarity, the colour coding is as follows: reviewers’ comments are shown in red, and our responses are shown in green.

REVIEWER 1

1. Remove the line “289 words” in the Abstract.

My comment: Done

2. Write the full scientific name when Apis first appears in the Abstract.

My comment: Done

3. The expression “beyond ~3–4 km” in the Abstract is incorrect; use either “beyond 3 km” or “beyond 4 km”.

My comment: I reworded this to: “These signals peaked within 1–3 km of nests, closely matching the effective foraging range of A. dorsata, whereas the distinction between nest-centred and random landscapes became less pronounced at larger distances.”

4. In the Introduction, the citation format “[1][2-4]” is wrong.

My comment: Done, Other formatting inconsistencies have also been corrected.

5. In the study area description, clearly provide the latitude and longitude range of the 1380 km² study area to facilitate reproducibility by other researchers.

My comment: In 2000, we mapped approximately 300 *Apis dorsata* colonies at 55 nesting sites across an area of 1380 km² in the central Chitwan plain (Fig. 1), bounded by the Narayani River at Meghauli in the west (84°7'3.70"E), the Rapti River in the south (27°28'26.64"N), the Narayani River near Bharatpur in the north (27°44'22.50"N), and east of the Tikauli Forest (84°38'41.84"E).

6. In the remote sensing data preprocessing section, supplement detailed information on cloud cover percentage and atmospheric correction method to improve *methodological completeness.*

My comment: In Methods we slightly extend the text to: “Land-use and land-cover (LULC) maps were generated using a hybrid classification strategy, because no single automated approach reliably separated all ecologically relevant classes across seasons. Rule-based classification was applied to spectrally distinct or temporally variable features, such as water bodies, river gravel, and clouds, whereas supervised and unsupervised spectral classification was used for heterogeneous land-use types. Manual digitisation was used where automated separation remained unreliable, particularly for cloud shadows. Clouds could be detected by scene-specific thresholding, but their shadows required manual delineation to prevent confusion with swamps, as both exhibited low reflectance.”

7. On page 9, for Eq.1 and Eq.2, add explanations for all symbols (e.g., ΔLUₙ₋ᵣ, Sₓ, M_c, P_s) when they first appear.

My comment: We have expanded the Methods section to include definitions and interpretative explanations and have revised the formatting of Equations 1 and 2.

8. In the caption of Figure 7, subscripts are missing for panels A–H.

My comment: The text runs such as: “Land-use categories are: (A) mustard/buckwheat, (B) fallow, (C) alluvial forest, (D) gravel/infrastructure, (E) wheat, (F) dry grass/open green, (G) rice, and (H) Sal forest.” This is correct without using subscripts as this is valid for both, December 1999 (panels 1) and February 2000 (panels 2) scenes. However, we have additionally revised the figure to improve clarity by applying distinct line thicknesses to horizontal and vertical elements. Please also note that we have harmonized the sequence and colouring of the land-use elements between Figs. 6 and 7.

9. In the crop rotation analysis of Figure 10, clarify whether the percentage is calculated relative to the total area of the corresponding crop or the total landscape area to avoid ambiguity.

My comment: The original caption reads: “Within a 4,500 m radius around a nest site, mustard occupied on average 8,750 ± 1,111 m². Of this area, 418 ± 75 m² (4.78%) was converted to buckwheat by February.” This wording makes clear that the areas of mustard and buckwheat are reported as absolute values in square metres, whereas the percentage in brackets refers specifically to the proportion of the average mustard area around each nest site that was subsequently occupied by buckwheat. The figure’s ordinate expresses relative rotation values as proportions of the total number of 15 × 15 m plots located between 100 and 4,500 m from each nest site. In Fig. 10, the secondary right-hand axis converts these proportions into corresponding land-use areas in square metres. This clarification should remove any remaining ambiguity regarding the scaling of the ordinate.

REVIEWER 2

We thank Reviewer 2 for the constructive comments. Below we respond to each point in detail.

1. My concern on the age of the data (1999–2000) is a significant point that requires a clear, formal address by the authors….

My comment:

While analysing the 1999–2000 data using the bee-eye perspective, we decided to revisit the Chitwan plains in 2026 to repeat the study as closely as possible. This renewed fieldwork was also facilitated by an invitation to give a keynote lecture at a honeybee congress in Rampur, Chitwan, in early April 2026. By the end of April, we had successfully returned from this expedition with valuable new data.

Today, satellite imagery (Landsat and Sentinel) is freely available and offers remarkably higher resolution than was possible in 2000, when we had to purchase three images at considerable cost. We therefore aim to repeat the study with a much denser temporal series, advanced image processing approaches, ideally using one or two images per month whenever suitable (almost cloud free) satellite coverage of the Chitwan area is available.

During the recent expedition, we surveyed an even larger area east, south and west of Bharatpur and documented how nesting conditions for Apis dorsata colonies have changed. However, analysing this extensive dataset will take at least six months.

One preliminary observation is that many buildings that formerly supported A. dorsata colonies have since been modernised and painted. This appears to have strongly reduced their suitability as nesting structures. At the same time, numerous water towers have been constructed across the region in a relatively regular spatial pattern, often in a uniform or even identical architectural design. These may now serve as useful proxies for assessing regional nesting-site availability and landscape attractiveness. Overall, our impression during this visit to Chitwan was that the population of Apis dorsata has declined markedly.

The satellite data will also allow us to test whether crop-rotation patterns and the agricultural mosaic have changed over the past 25 years. Based on our field impressions, we do not expect major changes, but this will have to be verified quantitatively.

For the Introduction part we add:

“We analyse a uniquely detailed dataset from 1999–2000 that combines field-based nest censuses with satellite-derived land-use information. While historical, this dataset captures colony–landscape relationships at a spatial resolution rarely available for large-scale pollinators and provides a baseline against which contemporary changes can be evaluated.”

In the Discussion part we add:

“We emphasise that our analysis is based on data from 1999 to 2000 and therefore represents a historical snapshot of colony landscape relationships rather than current conditions. However, the patterns identified here, particularly the spatially constrained signal precision structure at the foraging scale of Apis dorsata, reflect underlying behavioural and ecological mechanisms that are expected to be more stable than specific land use configurations. To assess their contemporary relevance, we conducted a new field campaign in 2026 and initiated a re analysis using high resolution, temporally dense satellite imagery, although these data are still under evaluation. The present study thus provides a quantitative baseline and methodological framework for testing the persistence of colony landscape coupling under ongoing environmental change.”

In the Abstract part we add: “While based on data from 1999–2000, this study provides a historical baseline of colony–landscape relationships, with ongoing work aimed at evaluating their persistence under current conditions.”

2. While the study notes that signals converge with random landscapes beyond ~3–4 km, it would be beneficial to further discuss potential factors beyond foraging range that might influence this boundary, such as environmental barriers or competition from other colonies.

My comment:

We agree that factors such as environmental barriers or neighbouring colonies may influence local foraging patterns. However, the observed convergence of the nest-centred signal towards zero beyond approximately 3–4 km is also expected from the structure of the analysis itself. With increasing radius, each circular sampling area includes a progressively larger portion of the regional landscape, so that local nest-associated deviations in land-use composition become increasingly diluted. When these values are averaged across all nesting sites, this dilution effect is further reinforced. Thus, convergence towards the random reference is a statistical expectation of the method, although local ecological factors may additionally modulate the precise distance at which this convergence occurs.

We add into Results: “This convergence is partly inherent to the radial sampling design, because increasing radius progressively incorporates more of the regional landscape matrix, thereby diluting local nest associated deviations from the random reference.”

And into Discussion we add: “Local ecological factors, including environmental barriers, colony density, or competition among neighbouring colonies, may nevertheless modulate the precise distance at which convergence occurs.”

3. If the authors have sufficiently addressed how these crops (mustard and buckwheat) specifically support the nutritional requirements (e.g., protein vs. carbohydrate balance) of the colonies during those specific seasons. I suggest a deeper integration of the nutritional quality of these floral resources would strengthen the discussion.

My comments:

We appreciate the reviewer’s suggestion regarding the nutritional aspects of floral resources. However, in the manuscript, we already emphasised that, within the Chitwan agricultural landscape, the only mass-flowering crops providing substantial forage for Apis dorsata are mustard (flowering approximately from November to late December) and, subsequently, buckwheat. These crops represent the dominant nectar and pollen sources during their respective flowering periods.

In contrast, the other major crops in the region—such as wheat, rice and maize—are wind-pollinated and do not provide meaningful forage for bees, which is a general pattern not only in Chitwan but globally.

We agree that a more explicit discussion of nutritional quality (e.g. protein vs. carbohydrate contributions) could strengthen the interpretation. However, given the strong limitation of available mass-flowering resources in this system, the key ecological driver is the temporal availability of these crops rather than differences in their nutritional composition.

We also note (while having visited Chitwan region again after 16 years) that recent land-use changes, particularly the expansion of banana plantations that may provide additional floral resources over extended periods, could become an important factor. This aspect will be addressed in more detail in our ongoing work.

We add the following sentences into Discussion

“The ecological relevance of mustard and buckwheat is not limited to their spatial association with nest sites, but also reflects their role as seasonal mass flowering resources. In the Chitwan crop mosaic, these crops provide both nectar and pollen during their respective flowering periods, whereas the dominant cereal crops, such as rice, wheat and maize, are wind pollinated and offer little direct forage for honey bees. Thus, in this system, the decisive factor is likely the seasonal availability of concentrated nectar and pollen resources rather than fine scale differences in nutritional composition among crop species.“

And also a final sentence:

“Future analyses should also consider whether newer land use elements, such as expanded banana cultivation, now provide additional forage outside the mustard and buckwheat flowering periods.”

4. Ensure the manuscript clearly justifies why these three specific snapshots (December 1999, February 2000, April 2000) are representative of the agricultural cycle, particularly regarding the potential for interannual variation in crop timing.

My comment:

In the paper, we have already explained why these three LANDSAT scenes were selected as representative time snapshots of the colonies’ seasonal stay in the Chitwan plains, including their reproductive phase within the annual cycle.

When the colonies arrived in Chitwan, mustard fields had already been prepared and sown, but flowering had only just begun, approximately two weeks after colony arrival. The December scene therefore captures the main mustard-flowering period.

After mustard harvest, and depending on the previous crop rotation, farmers shifted fields towards buckwheat, wheat or rice. The February scene documents the period when the Apis dorsata colonies reached the peak of their reproductive development, while buckwheat flowering provided an important floral resource.

By late February to mid-March, most fields had been harvested, with mainly wheat and rice still present. At this stage, colonies began to leave the plains and redistribute into surrounding forest areas. The April scene therefore represents the post-harvest situation, when only few colonies were still present in the Chitwan plains.

We add this as a compact paragraph in **Methods**, under image selection / temporal coverage:

The three LANDSAT scenes were selected to represent key phases of the seasonal stay of Apis dorsata colonies in the Chitwan plains. The December 1999 scene captures the main mustard flowering period shortly after colony arrival, whereas the February 2000 scene represents the peak reproductive phase of the colonies, when buckwheat flowering provided an important seasonal forage resource. The April 2000 scene documents the post harvest situation, when most mass flowering crops had disappeared and colonies had begun to leave the plains or redistribute into surrounding forest areas. Although interannual variation in crop timing is possible, these three scenes correspond to the dominant sequence of the regional agricultural cycle during the study season and were therefore used as representative temporal snapshots rather than as a continuous phenological record.

5. Reviewer’s statement: Provide a more explicit justification for using data from 1999–2000. Given the long gap between data collection and the current analysis, explain why these historical snapshots remain relevant and representative of the long-term spatial ecology of A. dorsata in this region.

My comment: Thank you for this important comment. We agree that the temporal gap between data collection and the present analysis requires explicit justification. Our intention is not to use the 1999–2000 dataset as a proxy for the current state of colony density or present-day land-use conditions in Chitwan. Rather, we use it as a historical baseline to analyse the spatial ecology of nest-site selection by Apis dorsata in relation to the seasonal landscape mosaic available at that time. Moreover, we can use the spectral information (derived from the satellite images in 1999-2000) as a land use land cover spectral library for future analyses.

We consider these data to remain relevant for three reasons. First, the dataset captures a biologically meaningful phase of the nesting season across several time points and includes a substantial number of nest sites, allowing spatial patterns to be evaluated at the landscape scale rather than from isolated observations. Second, the analysis is based on nest-centred versus random spatial contrasts, and thus aims to identify general relationships between colony placement and land-use structure rather than to describe only site-specific or short-lived conditions. Third, the historical character of the dataset is itself valuable, because it provides a baseline from a period before more recent landscape changes may have altered or obscured these relationships.

We have now clarified this rationale in the revised manuscript and explicitly state that these data are interpreted as evidence for general spatial associations and nest-positioning rules, not as a direct representation of the present-day status of the system. We also acknowledge that more recent data will be important for assessing temporal continuity and change.

We add this **short Methods paragraph**

The 1999 to 2000 dataset is used here as a historical baseline rather than as a proxy for present day colony density or current land use conditions in Chitwan. These data remain relevant because they combine a field based nest census with seasonally matched satellite scenes covering biologically distinct phases of the Apis dorsata nesting cycle. Our nest centred versus random comparison was designed to identify spatial associations between colony placement and the seasonal land use mosaic, rather than to describe only site specific conditions. We therefore interpret the results as evidence for colony landscape coupling during the 1999 to 2000 study period, while recognising that contemporary data are required to test whether these relationships persist today.

...and also this final sentence:

“Such contemporary validation is now underway based on renewed field surveys and higher resolution satellite imagery.”

6. Reviewer’s statement: Discuss the sensitivity of your null model approach. Specifically, address how the choice of "spatially constrained random reference landscapes" might influence the detection of signal precision at varying distances from the nest.

My comments: Thank you for this important point. The choice of spatially constrained random reference landscapes is indeed central to the interpretation of our null model. We used spatially constrained references deliberately, because the aim was not to compare nest sites with arbitrary locations across the entire region, but with locally available landscape alternatives within the same broader geographic context. This makes the null model more conservative and biologically more realistic, as it controls for large-scale geographic structure and asks whether colonies are associated with particular land-use configurations beyond what is locally available.

At the same time, we agree that the degree of spatial constraint can influence signal detection, especially across increasing distances from the nest. If random references were drawn from a much broader and more heterogeneous area, nest–random contrasts could become inflated by regional background differences. Conversely, stronger local constraint makes reference landscapes more similar to nest-centred landscapes and may therefore reduce contrast strength. Signal precision at larger radii is particularly sensitive to this issue, because larger buffers integrate more of the surrounding landscape and thus more strongly reflect the underlying spatial structure of the study region. We have clarified in the revised manuscript that our results should therefore be interpreted relative to this ecologically motivated and spatially constrained null model.

Our null model is designed to test whether nest-centred landscapes differ from plausible alternative landscapes available in the same region, not from the regional landscape in an unconstrained sense. This distinction is important, because the spatial constraint directly affects the variance structure of the reference set and therefore the apparent strength and precision of detected signals. In general, broader reference sampling would tend to increase contrast magnitude by incorporating more heterogeneous background conditions, whereas narrower local sampling provides a stricter test but may attenuate signal detection. This is particularly relevant at larger nest-centred radii, where both nest and reference landscapes increasingly integrate regional land-use structure. We now make this dependency explicit in the manuscript.

We add these sentences in the **Methods**:

“The null model was conditional on the use of spatially constrained random reference landscapes. This constraint was applied to compare nest centred landscapes with biologically plausible alternatives from the same geographic context, rather than with arbitrary locations across the full study region. Such spatial conditioning reduces the risk that apparent nest associated signals merely reflect large scale geographic gradients.”

We add these sentences in the **Discussion**:

“The degree of spatial constraint in the null model may influence the strength and precision of detectable contrasts. Broader reference sampling would likely increase contrast magnitude by incorporating more heterogeneous background landscapes, whereas tighter local sampling provides a more conservative test and may attenuate signal detection. This dependency is particularly relevant at larger radii around nests, where both nest centred and reference landscapes increasingly integrate regional spatial structure. Accordingly, the reported signal precision should be interpreted relative to this ecologically motivated null model.”

.. and this one sentence in the **Results**:

Estimates of signal precision are therefore conditional on the spatial definition of the reference landscapes.

Discussion

7. Reviewers remark: Since A. dorsata nesting is linked to resource availability, incorporates a more detailed discussion of the nutritional value of mustard and buckwheat.

My comments: While this point was already touched upon in the original manuscript, we agree that the functional significance of mustard and buckwheat as major seasonal floral resources for A. dorsata was not made explicit enough. We have therefore expanded the text to explain more clearly why these mass-flowering crops are likely to be nutritionally important and how their seasonal distribution may relate to nest-site positioning.

We add these sentences to the **Discussion** by replaceing the respective former paragraph:

“Mustard and buckwheat are not only conspicuous seasonal land use classes in the Chitwan plains, but also represent major floral resources for Apis dorsata during the nesting season. Their mass flowering provides spatially concentrated supplies of nectar and pollen, thereby combining carbohydrate and protein resources needed by large colonies with high foraging demand. By contrast, the dominant cereal crops in the region, including rice, wheat and maize, are wind pollinated and contribute little direct forage for honey bees. The observed nest associated patterns are therefore consistent with the interpretation that colony positioning is linked to the seasonal distribution of nutritionally rewarding forage resources.”

8. Reviewer’s remark: Connecting these crops to the specific physiological requirements of honey bees, especially during colony buildup or migration phases, would significantly enhance the ecological narrative.

My comment: We agree that the manuscript benefits from a more explicit connection between resource distribution and the physiological requirements of Apis dorsata colonies. We have therefore expanded the discussion to explain that mass-flowering crops such as mustard and buckwheat likely provide seasonally concentrated carbohydrate and pollen resources that are especially relevant during phases of colony buildup, brood production, and migratory settlement. This strengthens the ecological interpretation of the nest-associated land-use patterns.

However, to avoid repetitions I would **merge** the additional remarks into one stronger paragraph (compare to point 1 Discussion):

“Mustard and buckwheat are not only conspicuous seasonal land-use classes in the Chitwan plains, but also represent major functional forage resources for Apis dorsata during the nesting season. As mass-flowering crops, they may provide concentrated supplies of nectar and pollen during biologically critical phases, including colony establishment, comb construction, brood production and seasonal movement. Nectar supplies carbohydrates for flight and colony energetics, whereas pollen provides proteins and other nutrients required for brood development. By contrast, the dominant cereal crops in the region, including rice, wheat and maize, are wind-pollinated and contribute little direct forage for honey bees. The observed nest-associated patterns are therefore ecologically consistent with the physiological demands of large A. dorsata colonies, even though these demands were not measured directly in the present study.”

9. Reviewer’s remark: The current narrative assumes that crop mapping equates to resource availability, but it overlooks the critical nutritional balance required for A. dorsata physiology. I recommend the authors include a discussion on how the specific nutritional profiles of mustard and buckwheat might dictatecolony settlement.

My comment: We agree that crop mapping does not automatically equate to realised resource availability, and we now make this limitation more explicit in the manuscript. Our land-use analysis captures the spatial distribution of potential flowering resources at the landscape scale, but it does not directly measure floral reward quantity, phenological intensity, or the nutritional composition actually exploited by A. dorsata. We have therefore added a discussion noting that mustard and buckwheat may differ in the nectar and pollen profiles they provide, and that colony settlement is likely shaped not simply by crop presence, but by the seasonal availability of forage that meets the physiological and nutritional demands of the colony.

We add these sentences to the **Discussion**:

“Land use presence does not translate directly into realised resource availability. In our analysis, crop distribution therefore serves as a proxy for the spatial arrangement of potential forage resources at the landscape scale. The biological relevance of mustard and buckwheat may depend not only on their mapped extent, but also on flowering phenology, floral reward density, and the balance of nectar and pollen derived nutrients they provide. Our results should therefore be interpreted as consistent with a forage based settlement logic, while recognising that the nutritional quality of these resources was not measured directly in the present study.”

10. Reviewer’s remark: Specifically, do these crops provide a complementary nutritional sequence?

Finally, I suggest the following revised paragraph, which addresses several of the reviewer’s comments raised above while avoiding unnecessary repetition:

**“Mustard and buckwheat are not only conspicuous seasonal land-use classes in the Chitwan plains, but also represent major functional forage resources for** Apis dorsata **during the nesting season. As mass-flowering crops, they may provide concentrated supplies of nectar and pollen during biologically critical phases, including colony establishment, comb construction, brood production and seasonal movement. Moreover, mustard and buckwheat may form a temporally complementary sequence of flowering crops, potentially helping to sustain colony development across successive phases of the nesting season. Nectar supplies carbohydrates for flight and colony energetics, whereas pollen provides proteins and other nutrients required for brood development. By contrast, the dominant cereal crops in the region, including rice, wheat and maize, are wind-pollinated and contribute little direct forage for honey bees. The observed seasonal turnover from mustard to buckwheat is therefore ecologically consistent with the physiological demands of large** A. dorsata **colonies, even though nutritional complementarity and realised resource yield were not measured directly in the present study.”**

11. Reviewer’s remark: Addressing the nutritional quality of these floral resources would move the study from simple spatial correlation toward a deeper understanding of the ecological drivers of A. dorsata colony migration and site fidelity.

My comment: We agree that incorporating direct information on nutritional quality would move the interpretation towards a more mechanistic understanding of migration and site fidelity. At the same time, such an analysis would require data that are beyond the scope of the present study, which is focused on landscape structure and nest-associated spatial patterns. We therefore now clarify that our results identify spatial relationships consistent with resource-based colony settlement, while the nutritional mechanisms underlying these relationships remain to be tested directly.

We add these two sentences to the **Discussion**, and place them near the end of the forage resources paragraph:

“While our land use analysis identifies spatial patterns consistent with resource-based nest positioning, it does not directly resolve the nutritional mechanisms underlying colony migration or site fidelity. Addressing nectar and pollen quality in greater detail will be an important next step towards a more mechanistic understanding of these processes.”

12. Reviewer’s remark: Expand on why the signals converge with random landscapes beyond ~3–4 km. Clarify whether this distance boundary is solely a reflection of the effective foraging range or if it is also influenced by other landscape features or colony density dynamics.

My comment: We agree that the convergence beyond ~3–4 km should be interpreted with caution. While this distance is broadly consistent with an effective foraging scale for Apis dorsata, we do not interpret it as a purely behavioural boundary. At larger radii, nest-centred and random buffers increasingly sample the same background landscape, so local nest-associated signals become diluted by spatial averaging. We now clarify that the observed convergence likely reflects both biological scale and landscape-structural effects, and may also be influenced by colony-density-related spatial overlap.

We add these sentences to the **Discussion**:

“The convergence of nest associated signals with random landscapes beyond approximately 3 to 4 km is consistent with the idea that the strongest biologically relevant contrasts occur within the nearer foraging environment of colonies. However, this distance should not be interpreted as a strict foraging boundary alone. As buffer radius increases, both nest centred and random landscapes increasingly integrate the same regional land use matrix, so local nest associated contrasts are progressively diluted. The observed convergence therefore likely reflects a combination of biological scale, spatial averaging and broader landscape structure, and may also be influenced by overlap among neighbouring colony use areas.”
