# Supplementary material for: Nesting in anticipation: Spatial ecology of giant honey bees (Apis dorsata) in relation to crop succession mapped by remote sensing
Source: PLoS One. 2026 Jun 24;21(6):e0347045. doi: 10.1371/journal.pone.0347045 (PMC13293442; doi:10.1371/journal.pone.0347045)
Supplement: Peer review history

# Peer review: Decision Letter 0

**DOI:** 10.1371/journal.pone.0347045.r001

**Authors:** Olav Rueppell

28 Apr 2026

PONE-D-26-14865

Nesting in Anticipation: Spatial Ecology of Giant Honey Bees (Apis dorsata) in Relation to Crop Succession Mapped by Remote Sensing

PLOS One

Dear Dr. Kastberger,

Thank you for submitting your manuscript to PLOS ONE. After careful consideration, we feel that it has merit but does not fully meet PLOS ONE’s publication criteria as it currently stands. Therefore, we invite you to submit a revised version of the manuscript that addresses the points raised during the review process.

Both reviewers generally agree that this is a valuable study but make also valuable suggestions for improving the presentation.

Please submit your revised manuscript by Jun 12 2026 11:59PM. If you will need more time than this to complete your revisions, please reply to this message or contact the journal office at plosone@plos.org. When you're ready to submit your revision, log on to https://www.editorialmanager.com/pone/ and select the 'Submissions Needing Revision' folder to locate your manuscript file.

Please include the following items when submitting your revised manuscript:

- A letter that responds to each point raised by the academic editor and reviewer(s). You should upload this letter as a separate file labeled 'Response to Reviewers'.
- A marked-up copy of your manuscript that highlights changes made to the original version. You should upload this as a separate file labeled 'Revised Manuscript with Track Changes'.
- An unmarked version of your revised paper without tracked changes. You should upload this as a separate file labeled 'Manuscript'.

If you would like to make changes to your financial disclosure, please include your updated statement in your cover letter. Guidelines for resubmitting your figure files are available below the reviewer comments at the end of this letter.

If applicable, we recommend that you deposit your laboratory protocols in protocols.io to enhance the reproducibility of your results. Protocols.io assigns your protocol its own identifier (DOI) so that it can be cited independently in the future. For instructions see: https://journals.plos.org/plosone/s/submission-guidelines#loc-laboratory-protocols. Additionally, PLOS ONE offers an option for publishing peer-reviewed Lab Protocol articles, which describe protocols hosted on protocols.io. Read more information on sharing protocols at https://plos.org/protocols?utm_medium=editorial-email&utm_source=authorletters&utm_campaign=protocols.

As the corresponding author, your ORCID iD is verified in the submission system and will appear in the published article. PLOS supports the use of ORCID, and we encourage all coauthors to register for an ORCID iD and use it as well. Please encourage your coauthors to verify their ORCID iD within the submission system before final acceptance, as unverified ORCID iDs will not appear in the published article. *Only* the individual author can complete the verification step; PLOS staff *cannot* verify ORCID iDs on behalf of authors.

We look forward to receiving your revised manuscript.

Kind regards,

Olav Rueppell

Academic Editor

PLOS One

Journal Requirements:

When submitting your revision, we need you to address these additional requirements.

1. Please ensure that your manuscript meets PLOS ONE's style requirements, including those for file naming. The PLOS ONE style templates can be found at

https://journals.plos.org/plosone/s/file?id=wjVg/PLOSOne_formatting_sample_main_body.pdf and

https://journals.plos.org/plosone/s/file?id=ba62/PLOSOne_formatting_sample_title_authors_affiliations.pdf

2. Please note that PLOS One has specific guidelines on code sharing for submissions in which author-generated code underpins the findings in the manuscript. In these cases, we expect all author-generated code to be made available without restrictions upon publication of the work. Please review our guidelines at https://journals.plos.org/plosone/s/materials-and-software-sharing#loc-sharing-code and ensure that your code is shared in a way that follows best practice and facilitates reproducibility and reuse.

3. In your Methods section, please provide additional information regarding the permits you obtained for the work. Please ensure you have included the full name of the authority that approved the field site access and, if no permits were required, a brief statement explaining why.

4. Please update your submission to use the PLOS LaTeX template. The template and more information on our requirements for LaTeX submissions can be found at http://journals.plos.org/plosone/s/latex.

5. Thank you for stating the following in the Acknowledgments Section of your manuscript:

“This study was carried out in the framework of the Austrian Science Fund (FWF) project P13210, “Migratory Behaviour and Colony Aggregation in Giant Honeybees.” The publication costs for this article were funded by the University of Graz.”

We note that you have provided funding information that is not currently declared in your Funding Statement. However, funding information should not appear in the Acknowledgments section or other areas of your manuscript. We will only publish funding information present in the Funding Statement section of the online submission form.

Please remove any funding-related text from the manuscript and let us know how you would like to update your Funding Statement. Currently, your Funding Statement reads as follows:

“This study was carried out in the framework of the Austrian Science Fund (FWF) project P13210, “Migratory Behaviour and Colony Aggregation in Giant Honeybees.””

Please include your amended statements within your cover letter; we will change the online submission form on your behalf.

6. We note that the grant information you provided in the ‘Funding Information’ and ‘Financial Disclosure’ sections do not match.

When you resubmit, please ensure that you provide the correct grant numbers for the awards you received for your study in the ‘Funding Information’ section.

7. Thank you for stating the following financial disclosure:

“This study was carried out in the framework of the Austrian Science Fund (FWF) project P13210, “Migratory Behaviour and Colony Aggregation in Giant Honeybees.””

Please state what role the funders took in the study.  If the funders had no role, please state: "The funders had no role in study design, data collection and analysis, decision to publish, or preparation of the manuscript."

If this statement is not correct you must amend it as needed.

Please include this amended Role of Funder statement in your cover letter; we will change the online submission form on your behalf.

8. We note that Figures 1, 2, 3, 4, 5, 8, 9, S2 and S5 in your submission contain satellite images which may be copyrighted. All PLOS content is published under the Creative Commons Attribution License (CC BY 4.0), which means that the manuscript, images, and Supporting Information files will be freely available online, and any third party is permitted to access, download, copy, distribute, and use these materials in any way, even commercially, with proper attribution. For these reasons, we cannot publish previously copyrighted maps or satellite images created using proprietary data, such as Google software (Google Maps, Street View, and Earth). For more information, see our copyright guidelines: http://journals.plos.org/plosone/s/licenses-and-copyright.

We require you to either (1) present written permission from the copyright holder to publish these figures specifically under the CC BY 4.0 license, or (2) remove the figures from your submission:

1. You may seek permission from the original copyright holder of Figure(s) [#] to publish the content specifically under the CC BY 4.0 license.

We recommend that you contact the original copyright holder with the Content Permission Form (http://journals.plos.org/plosone/s/file?id=7c09/content-permission-form.pdf) and the following text:

“I request permission for the open-access journal PLOS ONE to publish XXX under the Creative Commons Attribution License (CCAL) CC BY 4.0 (http://creativecommons.org/licenses/by/4.0/). Please be aware that this license allows unrestricted use and distribution, even commercially, by third parties. Please reply and provide explicit written permission to publish XXX under a CC BY license and complete the attached form.”

Please upload the completed Content Permission Form or other proof of granted permissions as an "Other" file with your submission.

In the figure caption of the copyrighted figure, please include the following text: “Reprinted from [ref] under a CC BY license, with permission from [name of publisher], original copyright [original copyright year].”

2. If you are unable to obtain permission from the original copyright holder to publish these figures under the CC BY 4.0 license or if the copyright holder’s requirements are incompatible with the CC BY 4.0 license, please either i) remove the figure or ii) supply a replacement figure that complies with the CC BY 4.0 license. Please check copyright information on all replacement figures and update the figure caption with source information. If applicable, please specify in the figure caption text when a figure is similar but not identical to the original image and is therefore for illustrative purposes only.

The following resources for replacing copyrighted map figures may be helpful:

USGS National Map Viewer (public domain): http://viewer.nationalmap.gov/viewer/

The Gateway to Astronaut Photography of Earth (public domain): http://eol.jsc.nasa.gov/sseop/clickmap/

Maps at the CIA (public domain): https://www.cia.gov/library/publications/the-world-factbook/index.html and https://www.cia.gov/library/publications/cia-maps-publications/index.html

NASA Earth Observatory (public domain): http://earthobservatory.nasa.gov/

Landsat: http://landsat.visibleearth.nasa.gov/

USGS EROS (Earth Resources Observatory and Science (EROS) Center) (public domain): http://eros.usgs.gov/#

Natural Earth (public domain): http://www.naturalearthdata.com/

9. We note that Figures S1 and S3 in your submission contain copyrighted images. All PLOS content is published under the Creative Commons Attribution License (CC BY 4.0), which means that the manuscript, images, and Supporting Information files will be freely available online, and any third party is permitted to access, download, copy, distribute, and use these materials in any way, even commercially, with proper attribution. For more information, see our copyright guidelines: http://journals.plos.org/plosone/s/licenses-and-copyright.

We require you to either (1) present written permission from the copyright holder to publish these figures specifically under the CC BY 4.0 license, or (2) remove the figures from your submission:

1. You may seek permission from the original copyright holder of Figure(s) [#] to publish the content specifically under the CC BY 4.0 license.

We recommend that you contact the original copyright holder with the Content Permission Form (http://journals.plos.org/plosone/s/file?id=7c09/content-permission-form.pdf) and the following text:

“I request permission for the open-access journal PLOS ONE to publish XXX under the Creative Commons Attribution License (CCAL) CC BY 4.0 (http://creativecommons.org/licenses/by/4.0/). Please be aware that this license allows unrestricted use and distribution, even commercially, by third parties. Please reply and provide explicit written permission to publish XXX under a CC BY license and complete the attached form.”

Please upload the completed Content Permission Form or other proof of granted permissions as an "Other" file with your submission.

In the figure caption of the copyrighted figure, please include the following text: “Reprinted from [ref] under a CC BY license, with permission from [name of publisher], original copyright [original copyright year].”

2. If you are unable to obtain permission from the original copyright holder to publish these figures under the CC BY 4.0 license or if the copyright holder’s requirements are incompatible with the CC BY 4.0 license, please either i) remove the figure or ii) supply a replacement figure that complies with the CC BY 4.0 license. Please check copyright information on all replacement figures and update the figure caption with source information. If applicable, please specify in the figure caption text when a figure is similar but not identical to the original image and is therefore for illustrative purposes only.

10. If the reviewer comments include a recommendation to cite specific previously published works, please review and evaluate these publications to determine whether they are relevant and should be cited. There is no requirement to cite these works unless the editor has indicated otherwise.

11. Please review your reference list to ensure that it is complete and correct. If you have cited papers that have been retracted, please include the rationale for doing so in the manuscript text, or remove these references and replace them with relevant current references. Any changes to the reference list should be mentioned in the rebuttal letter that accompanies your revised manuscript. If you need to cite a retracted article, indicate the article’s retracted status in the References list and also include a citation and full reference for the retraction notice.

[Note: HTML markup is below. Please do not edit.]

Reviewers' comments:

Reviewer's Responses to Questions

**Comments to the Author**

1. Is the manuscript technically sound, and do the data support the conclusions?

The manuscript must describe a technically sound piece of scientific research with data that supports the conclusions. Experiments must have been conducted rigorously, with appropriate controls, replication, and sample sizes. The conclusions must be drawn appropriately based on the data presented.

Reviewer #1: Yes

Reviewer #2: Yes

**********

2. Has the statistical analysis been performed appropriately and rigorously?

Reviewer #1: Yes

Reviewer #2: Yes

**********

3. Have the authors made all data underlying the findings in their manuscript fully available?

The PLOS Data policy requires authors to make all data underlying the findings described in their manuscript fully available without restriction, with rare exception (please refer to the Data Availability Statement in the manuscript PDF file). The data should be provided as part of the manuscript or its supporting information, or deposited to a public repository. For example, in addition to summary statistics, the data points behind means, medians and variance measures should be available. If there are restrictions on publicly sharing data—e.g. participant privacy or use of data from a third party—those must be specified.

Reviewer #1: Yes

Reviewer #2: Yes

**********

4. Is the manuscript presented in an intelligible fashion and written in standard English?

PLOS ONE does not copyedit accepted manuscripts, so the language in submitted articles must be clear, correct, and unambiguous. Any typographical or grammatical errors should be corrected at revision, so please note any specific errors here.

Reviewer #1: Yes

Reviewer #2: Yes

**********

5. Review Comments to the Author

Please use the space provided to explain your answers to the questions above. You may also include additional comments for the author, including concerns about dual publication, research ethics, or publication ethics. (Please upload your review as an attachment if it exceeds 20,000 characters)

Reviewer #1: Seasonal migration and nesting of the giant honeybee Apis dorsata are widely associated with mass-flowering crops, yet how colony settlement relates to surrounding landscape structure remains poorly understood. This study analyzed land-use patterns within the effective foraging range of A. dorsata colonies in the central Chitwan plain (Nepal). The study is interesting with rich results, but there are errors in writing and presentation. I recommend that the manuscript undergo minor revisions to make the manuscript more clear.

1. Remove the line “289 words” in the Abstract.

2. Write the full scientific name when Apis first appears in the Abstract.

3. The expression “beyond ~3–4 km” in the Abstract is incorrect; use either “beyond 3 km” or “beyond 4 km”.

4. In the Introduction, the citation format “[1][2-4]” is wrong and should be revised to “[1-4]”.

5. In the study area description, clearly provide the latitude and longitude range of the 1380 km² study area to facilitate reproducibility by other researchers.

6. In the remote sensing data preprocessing section, supplement detailed information on cloud cover percentage and atmospheric correction method to improve methodological completeness.

7. On page 9, for Eq.1 and Eq.2, add explanations for all symbols (e.g., ΔLUₙ₋ᵣ, Sₓ, M_c, P_s) when they first appear.

8. In the caption of Figure 7, subscripts are missing for panels A–H.

9. In the crop rotation analysis of Figure 10, clarify whether the percentage is calculated relative to the total area of the corresponding crop or the total landscape area to avoid ambiguity.

Reviewer #2: Overall suggestion for improvement

1. My concern on the age of the data (1999–2000) is a significant point that requires a clear, formal address by the authors. In ecological studies, land-use dynamics are rarely static; therefore, relying on data from over 25 years ago without context or validation risks presenting findings that may not reflect contemporary ecological realities.

- My suggestion on this is authors may need to include a section in the "Discussion" or "Methods" that justifies why these specific years (1999–2000) remain representative. If this region has experienced minimal land-use change, they should provide evidence (e.g., historical land-cover maps or regional agricultural surveys) to support this claim.

- The authors may perform a "spot-check" validation using contemporary, high-resolution satellite imagery for a subset of the study area to see if the observed patterns of "signal-precision" hold true today.

- If full validation is not feasible, insist that the authors explicitly frame their work as a "historical analysis" rather than a current snapshot. They should clearly state the limitations of applying these findings to modern, potentially fragmented agricultural landscapes.

2. While the study notes that signals converge with random landscapes beyond ~3–4 km, it would be beneficial to further discuss potential factors beyond foraging range that might influence this boundary, such as environmental barriers or competition from other colonies.

3. If the authors have sufficiently addressed how these crops (mustard and buckwheat) specifically support the nutritional requirements (e.g., protein vs. carbohydrate balance) of the colonies during those specific seasons. I suggest a deeper integration of the nutritional quality of these floral resources would strengthen the discussion.

4. Ensure the manuscript clearly justifies why these three specific snapshots (December 1999, February 2000, April 2000) are representative of the agricultural cycle, particularly regarding the potential for interannual variation in crop timing.

Methodology

1. Provide a more explicit justification for using data from 1999–2000. Given the long gap between data collection and the current analysis, explain why these historical snapshots remain relevant and representative of the long-term spatial ecology of A. dorsata in this region.

2. Discuss the sensitivity of your null model approach. Specifically, address how the choice of "spatially constrained random reference landscapes" might influence the detection of signal precision at varying distances from the nest.

Discussion

1. Since A. dorsata nesting is linked to resource availability, incorporates a more detailed discussion of the nutritional value of mustard and buckwheat. Connecting these crops to the specific physiological requirements of honey bees, especially during colony buildup or migration phases, would significantly enhance the ecological narrative. The current narrative assumes that crop mapping equates to resource availability, but it overlooks the critical nutritional balance required for A. dorsata physiology. I recommend the authors include a discussion on how the specific nutritional profiles of mustard and buckwheat might dictate colony settlement. Specifically, do these crops provide a complementary nutritional sequence? Addressing the nutritional quality of these floral resources would move the study from simple spatial correlation toward a deeper understanding of the ecological drivers of A. dorsata colony migration and site fidelity

2. Expand on why the signals converge with random landscapes beyond ~3–4 km. Clarify whether this distance boundary is solely a reflection of the effective foraging range or if it is also influenced by other landscape features or colony density dynamics

**********

6. PLOS authors have the option to publish the peer review history of their article (what does this mean?). If published, this will include your full peer review and any attached files.

If you choose “no”, your identity will remain anonymous but your review may still be made public.

**Do you want your identity to be public for this peer review?** For information about this choice, including consent withdrawal, please see our Privacy Policy.

Reviewer #1: **Yes:**Xiao Chen

Reviewer #2: No

**********

[NOTE: If reviewer comments were submitted as an attachment file, they will be attached to this email and accessible via the submission site. Please log into your account, locate the manuscript record, and check for the action link "View Attachments". If this link does not appear, there are no attachment files.]

To ensure your figures meet our technical requirements, please review our figure guidelines: https://journals.plos.org/plosone/s/figures

You may also use PLOS’s free figure tool, NAAS, to help you prepare publication quality figures: https://journals.plos.org/plosone/s/figures#loc-tools-for-figure-preparation.

NAAS will assess whether your figures meet our technical requirements by comparing each figure against our figure specifications.

# Author comment: Author response to Decision Letter 1

**DOI:** 10.1371/journal.pone.0347045.r002

13 May 2026

Response to Editor and Reviewers

We thank the Editor for the careful evaluation of our manuscript. Below we respond to the Editor’s comments point by point (red: editor’s queries; green: my comments).

1. …recommend that you deposit your laboratory protocols in protocols.io

My comment: This study did not involve laboratory procedures; therefore, no lab protocols are available.

2. …code sharing for submissions in which author-generated code underpins the findings in the manuscript.

My comment: Data availability

For most figures and analytical chapters, the underlying data have been deposited in a form intended to enable independent verification and, to a large extent, recalculation of the reported results. The file naming scheme is designed so that the purpose of each file can be readily inferred from its name. Each dataset is accompanied by README documentation explaining its structure and relevance to the corresponding analyses. Where useful, analysis macros and scripts used in data processing, including Python, Fiji, and VBA code, are also provided as illustrative examples of the evaluation workflow. In a limited number of cases, however, full self-contained deposition is impractical because the datasets are unusually large or rely on complex cross-referencing among multiple Excel worksheets. In such cases, the relevant files and additional technical information are available from the first author upon reasonable request.

I have added the DOI for the Zenodo repository containing the supplementary data associated with each figure. This information is provided after the Acknowledgments, under the heading Data Availability:

“All figure-associated datasets, Excel workbooks, analytical macros, Python scripts, and supporting README files underlying this study are available from the Zenodo repository: https://doi.org/10.5281/zenodo.20157991”.

3. In your Methods section, please provide additional information regarding the permits you obtained for the work. Please ensure you have included the full name of the authority that approved the field site access and, if no permits were required, a brief statement explaining why.

My comment: We add this paragraph into Methods

Permits and field access

The study was based on non-invasive field observations and spatial analyses. No animals were collected, handled, or experimentally manipulated. Access to the relevant field sites was obtained with the agreement of landowners / local authorities / local communities / site managers. No specific formal permit was required for this work under the regulations applicable at the time of the study.

4. My comment: The manuscript has been prepared and submitted as a Word document, not as a LaTeX submission. Therefore, the PLOS LaTeX template is not applicable.

5. My comment: We have removed funding-related information from the Acknowledgments section, in accordance with PLOS ONE guidelines.

We request the Funding Statement to be updated as follows:

“This study was supported by the Austrian Science Fund (FWF) project P13210, ‘Migratory Behaviour and Colony Aggregation in Giant Honeybees.’ Publication costs were funded by the University of Graz.”

6. My comment: We have checked and harmonised the funding information across the ‘Funding Information’ and ‘Financial Disclosure’ sections. The entries now match and include the correct grant number (FWF project P13210) as well as publication funding by the University of Graz.

7. My comment: We request that the Funding Statement be updated as follows:

“This study was supported by the Austrian Science Fund (FWF) project P13210, ‘Migratory Behaviour and Colony Aggregation in Giant Honeybees.’ Publication costs were funded by the University of Graz.”

Role of the funders:

“The funders had no role in study design, data collection and analysis, decision to publish, or preparation of the manuscript.”

8. My comment: We have revised all figures listed by the editor (Figs. 1–5, 8, 9, S2 and S5) to ensure full compliance with PLOS ONE copyright and licensing requirements.

All background imagery is now derived exclusively from LANDSAT data (USGS/NASA; public domain) and has been processed and reclassified by the authors. The figures therefore represent analytical products and do not reproduce any original satellite imagery.

Any previously included third-party imagery (e.g. Google Earth) has been removed.

Accordingly, all figures constitute replacement figures that comply with the CC BY 4.0 license. Figure captions have been updated to include appropriate source attribution.

The analysis is based on LANDSAT source data, which are now publicly available through USGS archives. Equivalent scenes can be accessed using the scene identifiers and acquisition dates provided in the Methods section, ensuring reproducibility and transparency.

9. My comment: Supplementary Figures S1 and S3 are original photographs created by the first author (Gerald Kastberger), who is the copyright holder. These figures are now published under the Creative Commons Attribution License (CC BY 4.0). The figure captions have been updated accordingly.

No additional citation requests requiring action were identified.

10. My comment: We have reviewed the reference list and corrected minor inconsistencies and formatting issues to ensure completeness and accuracy.

My comment: Addendum

• We attach a corrected version of the manuscript in which the corrections are highlighted. Please note that this version does not yet fully reflect the latest status, as we have since completed a final polishing round. The final draft is now shorter than the version attached here.

• All figures have been checked and improved with respect to formatting, including line style, line thickness, and consistent text size.

• The file names have been selected to communicate the content of each figure clearly and concisely.

• Equations 1 and 2 were prepared using the Microsoft Word equation editor. We would prefer to retain this formatting in the manuscript.

• I have cited the Figures according your regulations e.g. “.. whereas the satellite perspective (Fig. 2B; Fig. 4C₁,C₄) reveals a strong association with the Narayani River and its floodplain. Grassland (Fig. 2E; Fig. 4D₂,D₃) and fallow land (S2f Fig; Fig. 4E₂,E₃)..”

We thank both reviewers for their constructive comments. Below, we respond to each point in detail. For clarity, the colour coding is as follows: reviewers’ comments are shown in red, and our responses are shown in green.

REVIEWER 1

1. Remove the line “289 words” in the Abstract.

My comment: Done

2. Write the full scientific name when Apis first appears in the Abstract.

My comment: Done

3. The expression “beyond ~3–4 km” in the Abstract is incorrect; use either “beyond 3 km” or “beyond 4 km”.

My comment: I reworded this to: “These signals peaked within 1–3 km of nests, closely matching the effective foraging range of A. dorsata, whereas the distinction between nest-centred and random landscapes became less pronounced at larger distances.”

4. In the Introduction, the citation format “[1][2-4]” is wrong.

My comment: Done, Other formatting inconsistencies have also been corrected.

5. In the study area description, clearly provide the latitude and longitude range of the 1380 km² study area to facilitate reproducibility by other researchers.

My comment: In 2000, we mapped approximately 300 Apis dorsata colonies at 55 nesting sites across an area of 1380 km² in the central Chitwan plain (Fig. 1), bounded by the Narayani River at Meghauli in the west (84°7'3.70"E), the Rapti River in the south (27°28'26.64"N), the Narayani River near Bharatpur in the north (27°44'22.50"N), and east of the Tikauli Forest (84°38'41.84"E).

6. In the remote sensing data preprocessing section, supplement detailed information on cloud cover percentage and atmospheric correction method to improve methodological completeness.

My comment: In Methods we slightly extend the text to: “Land-use and land-cover (LULC) maps were generated using a hybrid classification strategy, because no single automated approach reliably separated all ecologically relevant classes across seasons. Rule-based classification was applied to spectrally distinct or temporally variable features, such as water bodies, river gravel, and clouds, whereas supervised and unsupervised spectral classification was used for heterogeneous land-use types. Manual digitisation was used where automated separation remained unreliable, particularly for cloud shadows. Clouds could be detected by scene-specific thresholding, but their shadows required manual delineation to prevent confusion with swamps, as both exhibited low reflectance.”

7. On page 9, for Eq.1 and Eq.2, add explanations for all symbols (e.g., ΔLUₙ₋ᵣ, Sₓ, M_c, P_s) when they first appear.

My comment: We have expanded the Methods section to include definitions and interpretative explanations and have revised the formatting of Equations 1 and 2.

8. In the caption of Figure 7, subscripts are missing for panels A–H.

My comment: The text runs such as: “Land-use categories are: (A) mustard/buckwheat, (B) fallow, (C) alluvial forest, (D) gravel/infrastructure, (E) wheat, (F) dry grass/open green, (G) rice, and (H) Sal forest.” This is correct without using subscripts as this is valid for both, December 1999 (panels 1) and February 2000 (panels 2) scenes. However, we have additionally revised the figure to improve clarity by applying distinct line thicknesses to horizontal and vertical elements. Please also note that we have harmonized the sequence and colouring of the land-use elements between Figs. 6 and 7.

9. In the crop rotation analysis of Figure 10, clarify whether the percentage is calculated relative to the total area of the corresponding crop or the total landscape area to avoid ambiguity.

My comment: The original caption reads: “Within a 4,500 m radius around a nest site, mustard occupied on average 8,750 ± 1,111 m². Of this area, 418 ± 75 m² (4.78%) was converted to buckwheat by February.” This wording makes clear that the areas of mustard and buckwheat are reported as absolute values in square metres, whereas the percentage in brackets refers specifically to the proportion of the average mustard area around each nest site that was subsequently occupied by buckwheat. The figure’s ordinate expresses relative rotation values as proportions of the total number of 15 × 15 m plots located between 100 and 4,500 m from each nest site. In Fig. 10, the secondary right-hand axis converts these proportions into corresponding land-use areas in square metres. This clarification should remove any remaining ambiguity regarding the scaling of the ordinate.

REVIEWER 2

We thank Reviewer 2 for the constructive comments. Below we respond to each point in detail.

1. My concern on the age of the data (1999–2000) is a significant point that requires a clear, formal address by the authors….

My comment:

While analysing the 1999–2000 data using the bee-eye perspective, we decided to revisit the Chitwan plains in 2026 to repeat the study as closely as possible. This renewed fieldwork was also facilitated by an invitation to give a keynote lecture at a honeybee congress in Rampur, Chitwan, in early April 2026. By the end of April, we had successfully returned from this expedition with valuable new data.

Today, satellite imagery (Landsat and Sentinel) is freely available and offers remarkably higher resolution than was possible in 2000, when we had to purchase three images at considerable cost. We therefore aim to repeat the study with a much denser temporal series, advanced image processing approaches, ideally using one or two images per month whenever suitable (almost cloud free) satellite coverage of the Chitwan area is available.

During the recent expedition, we surveyed an even larger area east, south and west of Bharatpur and documented how nesting conditions for Apis dorsata colonies have changed. However, analysing this extensive dataset will take at least six months.

One preliminary observation is that many buildings that formerly supported A. dorsata colonies have since been modernised and painted. This appears to have strongly reduced their suitability as nesting structures. At the same time, numerous water towers have been constructed across the region in a relatively regular spatial pattern, often in a uniform or even identical architectural design. These may now serve as useful proxies for assessing regional nesting-site availability and landscape attractiveness. Overall, our impression during this visit to Chitwan was that the population of Apis dorsata has declined markedly.

The satellite data will also allow us to test whether crop-rotation patterns and the agricultural mosaic have changed over the past 25 years. Based on our field impressions, we do not expect major changes, but this will have to be verified quantitatively.

For the Introduction part we add:

“We analyse a uniquely detailed dataset from 1999–2000 that combines field-based nest censuses with satellite-derived land-use information. While historical, this dataset captures colony–landscape relationships at a spatial resolution rarely available for large-scale pollinators and provides a baseline against which contemporary changes can be evaluated.”

In the Discussion part we add:

“We emphasise that our analysis is based on data from 1999 to 2000 and therefore represents a historical snapshot of colony landscape relationships rather than current conditions. However, the patterns identified here, particularly the spatially constrained signal precision structure at the foraging scale of Apis dorsata, reflect underlying behavioural and ecological mechanisms that are expected to be more stable than specific land use configurations. To assess their contemporary relevance, we conducted a new field campaign in 2026 and initiated a re analysis using high resolution, temporally dense satellite imagery, although these data are still under evaluation. The present study thus provides a quantitative baseline and methodological framework for testing the persistence of colony landscape coupling under ongoing environmental change.”

In the Abstract part we add: “While based on data from 1999–2000, this study provides a historical baseline of colony–landscape relationships, with ongoing work aimed at evaluating their persistence under current conditions.”

2. While the study notes that signals converge with random landscapes beyond ~3–4 km, it would be beneficial to further discuss potential factors beyond foraging range that might influence this boundary, such as environmental barriers or competition from other colonies.

My comment:

We agree that factors such as environmental barriers or neighbouring colonies may influence local foraging patterns. However, the observed convergence of the nest-centred signal towards zero beyond approximately 3–4 km is also expected from the structure of the analysis itself. With increasing radius, each circular sampling area includes a progressively larger portion of the regional landscape, so that local nest-associated deviations in land-use composition become increasingly diluted. When these values are averaged across all nesting sites, this dilution effect is further reinforced. Thus, convergence towards the random reference is a statistical expectation of the method, although local ecological factors may additionally modulate the precise distance at which this convergence occurs.

We add into Results: “This convergence is partly inherent to the radial sampling design, because increasing radius progressively incorporates more of the regional landscape matrix, thereby diluting local nest associated deviations from the random reference.”

And into Discussion we add: “Local ecological factors, including environmental barriers, colony density, or competition among neighbouring colonies, may nevertheless modulate the precise distance at which convergence occurs.”

3. If the authors have sufficiently addressed how these crops (mustard and buckwheat) specifically support the nutritional requirements (e.g., protein vs. carbohydrate balance) of the colonies during those speci

Attachment Submitted filename: Rebuttal letter 1abcd - Response to Reviewers.docx

# Editor report: Decision Letter 1

**DOI:** 10.1371/journal.pone.0347045.r003

**Authors:** Olav Rueppell

22 May 2026

Nesting in Anticipation: Spatial Ecology of Giant Honey Bees (Apis dorsata) in Relation to Crop Succession Mapped by Remote Sensing

PONE-D-26-14865R1

Dear Dr. Kastberger,

We’re pleased to inform you that your manuscript has been judged scientifically suitable for publication and will be formally accepted for publication once it meets all outstanding technical requirements.

Within one week, you’ll receive an e-mail detailing the required amendments. When these have been addressed, you’ll receive a formal acceptance letter and your manuscript will be scheduled for publication.

An invoice will be generated when your article is formally accepted. Please note, if your institution has a publishing partnership with PLOS and your article meets the relevant criteria, all or part of your publication costs will be covered. Please make sure your user information is up-to-date by logging into Editorial Manager at Editorial Manager® and clicking the ‘Update My Information' link at the top of the page. For questions related to billing, please contact billing support.

If your institution or institutions have a press office, please notify them about your upcoming paper to help maximize its impact. If they’ll be preparing press materials, please inform our press team as soon as possible -- no later than 48 hours after receiving the formal acceptance. Your manuscript will remain under strict press embargo until 2 pm Eastern Time on the date of publication. For more information, please contact onepress@plos.org.

Kind regards,

Olav Rueppell

Academic Editor

PLOS One

Additional Editor Comments (optional):

Reviewers' comments:

# Editor report: Acceptance letter

**DOI:** 10.1371/journal.pone.0347045.r004

**Authors:** Olav Rueppell

PONE-D-26-14865R1

PLOS One

Dear Dr. Kastberger,

I'm pleased to inform you that your manuscript has been deemed suitable for publication in PLOS One. Congratulations! Your manuscript is now being handed over to our production team.

At this stage, our production department will prepare your paper for publication. This includes ensuring the following:

* All references, tables, and figures are properly cited

* All relevant supporting information is included in the manuscript submission,

* There are no issues that prevent the paper from being properly typeset

You will receive further instructions from the production team, including instructions on how to review your proof when it is ready. Please keep in mind that we are working through a large volume of accepted articles, so please give us a few days to review your paper and let you know the next and final steps.

Lastly, if your institution or institutions have a press office, please let them know about your upcoming paper now to help maximize its impact. If they'll be preparing press materials, please inform our press team within the next 48 hours. Your manuscript will remain under strict press embargo until 2 pm Eastern Time on the date of publication. For more information, please contact onepress@plos.org.

You will receive an invoice from PLOS for your publication fee after your manuscript has reached the completed accept phase. If you receive an email requesting payment before acceptance or for any other service, this may be a phishing scheme. Learn how to identify phishing emails and protect your accounts at https://explore.plos.org/phishing.

If we can help with anything else, please email us at customercare@plos.org.

Thank you for submitting your work to PLOS ONE and supporting open access.

Kind regards,

PLOS ONE Editorial Office Staff

on behalf of

Dr. Olav Rueppell

Academic Editor

PLOS One
